# Conjugates of desferrioxamine with 5-aminolevulinic or γ-aminobutyric acid: synthesis, theoretical studies, iron-chelating ability, and penetration in human Caco-2 cells

**DOI:** 10.1007/s10534-026-00841-7

**Published:** 2026-06-24

**Authors:** Ernani Lacerda, Vasilii D. Khripun, M. Teresa Machini, Breno Pannia Espósito

**Affiliations:** 1 Department of Fundamental Chemistry, Institute of Chemistry, University of São Paulo, São Paulo, SP Brazil; 2 Federal Institute of Education, Science and Technology of Bahia, Jacobina, BA Brazil; 3 Institute of Chemistry, Federal University of Mato Grosso do Sul, Campo Grande, MS Brazil; 4 Department of Biochemistry, Institute of Chemistry, University of São Paulo, São Paulo, SP Brazil

**Keywords:** Iron overload, Iron-chelating conjugates, Aminoacyl-desferrioxamine, Cell permeability, DFT calculations

## Abstract

Iron overload (IO) is a harmful condition in humans that promotes oxidative damage to DNA and lipids. As IO has been associated with the onset of neurodegenerative diseases (NDs), chelation therapy has been extensively used for treating IO and considered potentially suitable for NDs treatment. Desferrioxamine (DFO) presents high affinity and selectivity for iron(III), however, its clinical use as chelator is limited due to its low cell permeability. To circumvent this limitation, synthetic routes and conditions were selected for conjugating DFO to 5-aminolevulinic acid (5-ALA) or γ-aminobutyric acid (GABA), non-proteinogenic amino acids previously reported as permeable to the blood–brain barrier (BBB). Once obtained and purified, the conjugates 5-ALA-DFO and GABA-DFO were characterized by LC/ESI–MS and ^1^H and ^13^C NMR. Both exhibited iron binding abilities comparable to that of DFO, displayed identical antioxidant activity, weak interactions with human serum albumin, and were unable to remove iron from transferrin (Tf). Based on DFT calculations, a theoretical model was developed that allows to predict the efficiency of iron chelation with DFO and its conjugates, in good agreement with experimental results. In assays with human colorectal adenocarcinoma (Caco-2) cells, a model for the blood–brain barrier (BBB), both chelators were non-toxic, and unlike DFO or 5-ALA-DFO, GABA-DFO was able to bind intracellular iron. Altogether, these results demonstrate that the chemical conjugation proposed here was highly effective and that GABA-DFO is a promising intracellular iron chelator.

**Supplementary Information:**

The online version contains supplementary material available at 10.1007/s10534-026-00841-7.

## Introduction

Iron is indispensable for life and is the most abundant transition metal in the human body. It is a cofactor of several biomolecules and, due to the biologically accessible interconversion between ferric, Fe(III), and ferrous, Fe(II), forms, participates in a wide range of processes, such as oxygen transport, synthesis of DNA, antioxidant defenses, and electron transport chain. Iron transport, uptake, efflux, and storage are tightly regulated by proteins such as transferrin (Tf), divalent metal transporter 1, ferroportin, and ferritin, respectively. These proteins also determine the cytosolic levels in the labile iron pool (LIP), the main source of intracellular reactive iron (Espósito et al. 2003; Grotto 2008). Iron overload can disrupt this balance by increasing the production of reactive oxygen species (ROS), such as hydroxyl radicals, and by promoting oxidative damage to DNA and lipids. This condition has been related to the onset of neurodegenerative diseases (NDs) and cancer (Joppe et al. 2019; Lee and Kovacs 2024; Mazur et al. 2024; Piperno et al. 2020; Szlasa et al. 2022).

Chelation therapy is one of the possible treatments for iron overload (IO), and three chelators are currently approved for clinical use: desferrioxamine (DFO), deferiprone (DFP), and deferasirox. Some of the requirements for a chelating agent to be used for IO include selectivity to the metal, high stability of the complex formed, low cost, low reactivity of the Fe-chelator complex towards biological reducing and oxidizing agents, good absorption, and bioavailability at the biological target (Jones et al. 2020; Nurchi et al. 2016).

**Fig. 1:**
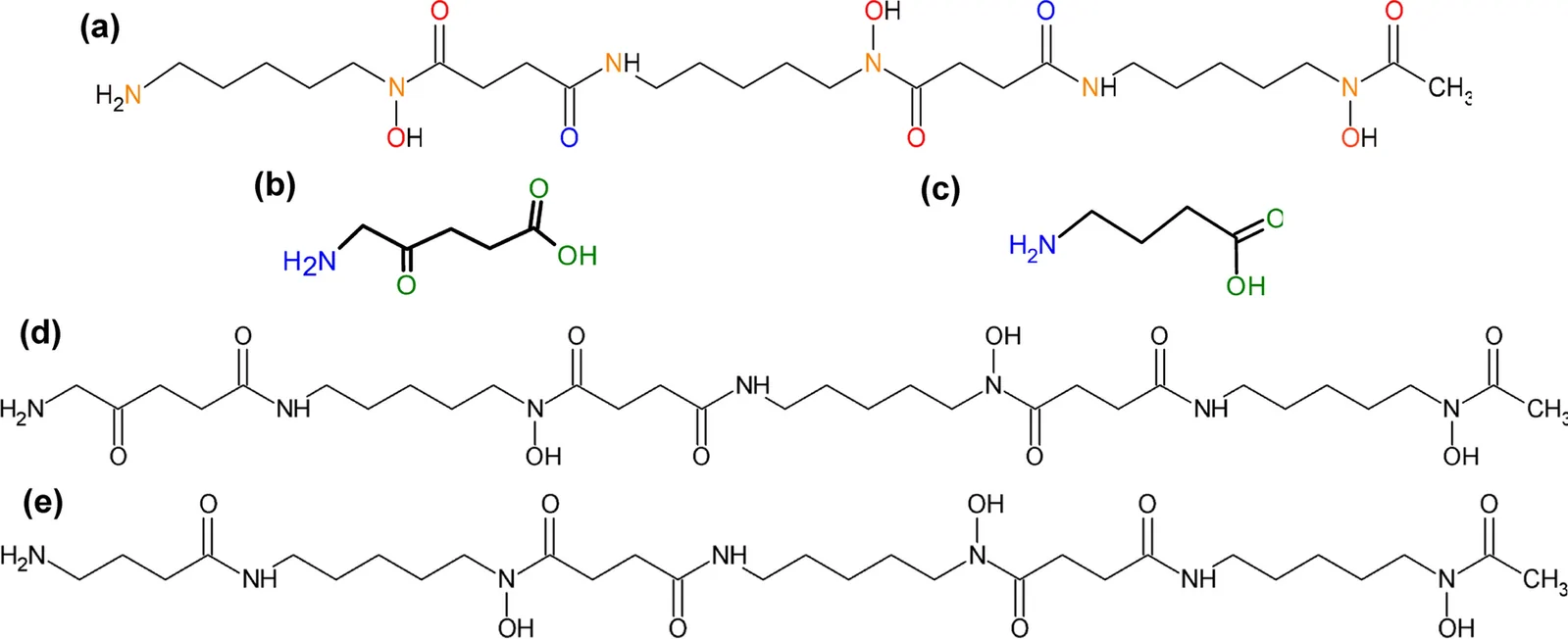
Structural formulas of a DFO, b 5-ALA, c GABA, d 5-ALA-DFO, and e GABA-DFO

Among the clinically used chelators, the bacterial siderophore DFO (Fig. 1a) is the only hexadentate one, forming a highly stable (logK_FeDFO_ = 30.5) and redox-inactive ferric complex (Hider and Kong 2010). Despite its high affinity and selectivity for iron(III) (Crisponi et al. 2015), DFO is a hydrophilic chelator with low permeability across physiological barriers, including the BBB (Farr and Xiong 2021; Jones et al. 2020). Even with these limitations, DFO has shown promising results for inducing apoptosis in gastric cancer and acute lymphoblastic leukemia cells (Kim et al. 2016; You et al. 2022). This molecule also promoted a successive decrease in Alzheimer’s Disease-associated dementia (McLachlan et al. 1991), and stabilized symptoms in 70% of multiple sclerosis patients after 12 months of treatment (Dusek et al. 2016).

Given these potential applications of DFO, new strategies have been created to enhance its permeability across physiological barriers. Our research groups have been developing conjugates of DFO with molecules able to interact with cell membranes and the BBB. In fact, cell-penetrating and mitochondria-penetrating peptides (CPP and MPP, respectively) (Goswami et al. 2014; Alta et al. 2017), and small molecules, such as caffeine (Alta et al. 2014) or aromatic amines (Carvalho et al. 2021), have been conjugated with DFO. Inspired by the fact that 5-aminolevulinic acid (5-ALA) and γ-aminobutyric acid (GABA) (Fig. 1b, c) cross the BBB, in this work we designed, synthesized and studied conjugates of DFO with these non-proteinogenic amino acids as possible facilitators of the siderophore transport across this important physiological barrier.

GABA is an inhibitory neurotransmitter in CNS, whose deficiency has been associated with some NDs and mental disorders. Since GABA efflux through BBB is greater than its influx (Kakee et al. 2001), a conjugate of DFO with GABA could permeate BBB more efficiently, bind iron and remove it from animal brain. Besides, it is known that conjugates or derivatives of this amino acid with prostaglandin E_2_ (E_2_-GABA) and arachidonic ester (AA-GABA), respectively, selectively interact with the GABA_A_-receptor. Also, depending on the form of GABA (carboxyl or ester form), the conjugate or derivative produced could show cerebrovascular activity on rats with ischemic brain injury (Gan’shina et al. 2024). Regarding 5-ALA, this is the precursor of protoporphyrin IX (PpIX) in mitochondria and is used for the detection and photodestruction of brain tumors during surgery. Having high permeability through the blood-tumor barrier, 5-ALA can interact with a range of transporters present in the human brain, such as those able to mediate the entry of GABA, glutamate or tripeptides, a factor that may facilitate its BBB permeability (Novotny and Stummer 2003).

In this study, we synthesized, purified and characterized the new low molar-weight conjugates 5-ALA-DFO and GABA-DFO (Fig. 1d, e), then proceeded to study their interactions with plasmatic proteins (serum albumin, transferrin), their binding with iron(III), and antioxidant activities. Finally, we examined the penetrating abilities of the new conjugates in Caco-2 cells.

## Materials and methods

### Chemicals

All chemicals were used without further purification. 5-(Boc-amino)levulinic acid was purchased from Ambeed (USA). Desferrioxamine mesylate (DFO; Cristália, Brazil) was a gift from fhe Brazilian Thalassemia Association (ABRASTA). 1-ethyl-3-(3-dimethylaminopropyl)carbodiimide (EDC), analytical TLC plates pre-coated with silica gel, ascorbic acid, dichloromethane, and trifluoroacetic acid were obtained from Merck (Germany). 3-(4,5-Dimethyl-2-thiazolyl)−2,5-diphenyl-2H-tetrazolium bromide (MTT), Dulbecco’s Modified Eagle Medium (DMEM) high glucose, fetal bovine serum (FBS), penicillin/streptomycin, and trypsin–EDTA (1 ×) 0.25% were purchased from Gibco (USA). 5-([4,6-dichorotriazin-2-yl]amino)fluorescein hydrochloride was purchased from Invitrogen. Calcein-AM and HEPES were obtained from ThermoFisher (USA). Acetic acid, chloroform, ethanol, and methanol were purchased from Synth (Brazil). Diethyl ether and sodium nitrilotriacetate were purchased from Vetec (Brazil). Dimethylamine (DMA) 40% was purchased from Seelze-Hannover (Germany). Dihydrorhodamine (DHR) chloride was purchased from Biothium (USA). 1-hydroxybenzotriazol (HOBt), 4-(Fmoc-amino)butyric acid, acetonitrile (ACN), DMF, DMSO, DMSO-d_6_, calcein, HEPES, ferrous ammonium sulfate (FAS) were obtained from Sigma-Aldrich (USA).

### Instruments

Elemental analysis was performed in a CHN 2400 (Perkin Elmer, Waltham, USA) equipment. Melting points were determined in a Kofler Apparatus (Fisher Scientific, Loughbourough, United Kingdom). FTIR spectra were obtained with a Cary 630 spectrometer (Agilent, Santa Clara, USA) equipped with Attenuated Total Reflectance (ATR) accessory. Fluorescence and electronic absorbance spectra were obtained in a SpectraMax M4 (Molecular Devices, Sunnyvale, USA) or FluoStar Optima (BMG, Ortenberg, Germany) microplate reader. NMR spectra were recorded in a Varian Inova (Oklahoma, USA) or Bruker AIII (Bruker, Billerica, USA) spectrometer operating at 300 MHz and 75 MHz for ^1^H and ^13^C, respectively. When purification was done by preparative reverse-phase HPLC, a RP-HPLC 600E system from Waters (Millford, MA, USA) containing a Waters Delta 600 pump, Waters 2487 Dual Absorbance detector, a Rheodyne 3725–119 manual injector, a Kipp & Zonen SE 124 recorder and a Vydac C18 (10 µm, 300 Å, 2.2 × 25 cm) column were used. Analysis by LC/ESI–MS were performed using a Shimadzu Liquid Chromatographer (Kyoto, Japan) composed of two pumps (LC-20AD), a DGU-20A_3_ degasser, a Rheodyne 8125 manual injector, a CTO-20A column oven, a C18 Shim-pack GVP-ODS precolumn and a C18 Shim-pack GVP-ODS column, and SDP-20AV detector coupled to an AmaZon X mass spectrometer (Bruker Daltonics, Fahrenheitstrasse, Germany). The system employs the software HyStar 3.2 for analyzing the mass spectra obtained. Fluorescence emission spectra were recorded using a 450 W Xenon lamp as excitation source, coupled to a Horiba Jobin Yvon Fluorolog-3 with a simple monochromator Spex 1680 and a photomultiplier R928P PMT. Circular Dichroism spectra were obtained in a Jasco J720 spectrophotometer using a source light of Xe. Fluorescence microscopy employed a Leica DMi8 Fluorescence Microscope with FITC (excitation: 460–500 nm; DC: 505 nm; emission: 512–542 nm) fluorescence filter cube. The images were recorded using a DFC 7000 GT camera with 20 × magnitude, and treated with the software LASX.

### Synthesis and characterization of the conjugates

Both compounds were synthesized in two steps (Alta et al. 2014; Jad et al. 2015; Loffredo et al. 2009; Miranda et al. 1986): formation of peptide bond at Boc-5-ALA-DFO and Fmoc-GABA-DFO followed by the removal of Fmoc or Boc in order to obtain the conjugates 5-ALA-DFO and GABA-DFO. All reactions were monitored by TLC using chloroform: methanol: acetic acid (85:10:5, v/v/v) as a mobile phase, silica as stationary phase, and the solution 0.28 M ninhydrin in ethanol as color developer spray. The products of each step were also analyzed by LC/ESI–MS. For LC, an aliquot of 5 µL of each product in DMSO or 60% ACN/0.09% TFA/H_2_O at the concentration of 1 mg/mL was injected, and the elution was done using a linear gradient of B (where A was 0.1% TFA and B was 60% ACN/0.09% TFA/H_2_O, 5–95% for 30 min) at flow rate of 0.8 mL/min with detection at 210 nm.

*Boc-5-ALA-DFO:* 636 mg (0.96 mmol) of DFO and 135 μL (0.96 mmol) of triethylamine (TEA) were mixed in 8.2 mL of mixture ethanol:DMF (1:1, v/v). Then 200 mg (0.88 mmol) of Boc-5-aminolevulinic acid (Boc-5-ALA), 199.2 mg (1.04 mmol) of EDC and 134.4 mg (0.88 mmol) of HOBt were dissolved in same volume of mixture ethanol:DMF (1:1, v/v). The system was kept under heating at 40 °C and stirring for 24 h. The resulting mixture was centrifuged, and a yellow solid was washed with water, methanol and diethyl ether, and then dried under vacuum overnight.

Yield: 21.3%. m.p.: 148.3–150.0 °C. R_f_: 0.50 (negative to ninhydrin test). ^1^H-NMR (300 MHz, dmso-d_6_): 9.61 (s, 3H), 7.78–7.92 (m, 3H), 7.02–7.14 (m, 1H), 3.70–3.83 (m, 2H), 3.45–3.50 (m, 6H), 2.94–3.10 (m, 6H), 2.55–2.68 (m, 6H), 2.23–2.37 (m, 6H), 1.99 (s, 3H), 1.47–1.59 (m, 6H), 1.31–1.47- (m, 15H), 1.17–1.29 (m, 6H). ^13^C NMR (75 MHz, dmso-d_6_): 207.02, 172.45, 171.80, 171.30, 170.63, 156.25, 78.54, 50.02, 47.55, 47.26, 38.90, 34.73, 30.38, 29.29, 28.65, 28.04, 26.51, 23.97, 20.83. ESI–MS (m/z): % theoretical (% experimental): 774.92 (774.57). Elemental analysis: % theoretical (% experimental) for C_35_H_63_N_7_O_12_ (773.93 g.mol^−1^): C = 54.32% (54.11%), H = 8.21% (7.83%), N = 12.67% (12.74%).

*5-ALA-DFO:* 150 mg of Boc-5-ALA-DFO (0.22 mmol) was dissolved in mixture 1:1 (v/v) of dichloromethane and trifluoroacetic acid (37.6 mL) at room temperature for 30 min. After removing the solvent under high pressure, 9 mL of methanol was added to the flask and the mixture was kept at 4 °C overnight. A yellow solid was separated through centrifugation, washed with methanol and cold diethyl ether and dried under vacuum. The crude product was purified by preparative reverse-phase HPLC using two eluents: A (trifluoroacetic acid 0.1% in water) and B (40% acetonitrile, 0.09% trifluoroacetic acid in water). A sample of 87 mg of crude 5-ALA-DFO was dissolved in 1 mL of DMSO and the solution was loaded following the sequence: 5% A for 5 min, 10% B for 10 min, linear gradient of 10–60% B in 90 min, and 95% B for 10 min. The fractions collected were analyzed by ESI–MS (direct infusion) and the ones containing 5-ALA-DFO were pooled, lyophilized and weighted.

Yield: 30.3%. m.p.: 152.5–154.1 °C. R_f_: 0 (positive to ninhydrin test). ^1^H-NMR (300 MHz, dmso-d_6_): 9.64 (m, 3H), 7.99 (s, 3H), 7.85–7.93 (m, 1H), 7.79 (t, J = 6 Hz, 2H), 6.60 (s, 0.5H), 3.96 (s, 1H), 3.45 (t, J = 6 Hz, 6H), 2.95–3.04 (m, 6H), 2.68 (t, J = 6 Hz, 1H), 2.53–2.63 (m, 4H), 2.38 (t, J = 6 Hz, 2H), 2.23–2.33 (m, 6H), 1.96 (s, 3H), 1.45–1.55 (m, 6H), 1.31–1.42 (m, 6H), 1.15–1.26 (m, 6H). ^13^C NMR (75 MHz, dmso-d_6_): (75 MHz, dmso-d_6_): 203.89, 173.87, 172.42, 171.77,170.99, 170.60, 88.76, 47.55, 47.29, 44.57, 38.90, 35.08, 30.86, 30.35, 29.29, 28.02, 26.51, 23.96, 20.83. ESI–MS (m/z): % theoretical (% experimental): 674.80 (674.43). Elemental analysis: % theoretical (% experimental) for (C_30_H_56_N_7_O_10_) (CF_3_COO) 0.2 H_2_O (823.86 g.mol^−1^): C = 46.65% (46.74%), H = 7.34% (6.95%), N = 11.90% (11.45%).

*Fmoc-GABA-DFO:* 715 mg (1.08 mmol) of DFO, 152 μL (1.08 mmol) of TEA, 322 mg (0.99 mmol) of Fmoc-GABA, 230 mg (1.2 mmol) of EDC and 134 mg (0.99 mmol) of HOBt were mixed in 13 mL of DMF for 4.5 h under heating at 50 °C and stirring. A white solid was formed, filtered, washed with DMF, water, methanol and diethyl ether, and dried under vacuum. The reaction was also conducted in 1:1 (v/v) ethanol:DMF using the same proportions of reagents, but for 16 h at 40 °C. The same results were obtained.

Yield: 54.3%. m.p.: 148.8–149.6. R_f_: 0.60 (negative to ninhydrin test). ^1^H-NMR (300 MHz, dmso-d_6_): 9.62 (s, 1H), 7.91 (d, J = 7.2 Hz, 2H), 7.77–7.88 (m, 3H), 7.70 (d, J = 7.2 Hz, 1H), 7.43 (t, J = 7.2 Hz, 2H), 7.35 (m, 2H), 6.30 (s, 1H), 4.18–4.35 (m, 2H), 3.45–3.52 (m, 8H), 2.97–3.05 (q, 6H), 2.59 (t, J = 6.9 Hz, 4H), 2.28 (t, J = 6.9 Hz, 4H), 2.06 (t, J = 6.9 Hz, 2H), 1.98 (s, 3H), 1.57–1.69 (qn, J = 6.9 Hz, 2H), 1.45–1.57 (m, 6H), 1.32–1.44 (m, 6H), 1.16–1.29 (m, 6H). ^13^C NMR (75 MHz, dmso-d_6_): 172.43, 172.14, 171.80, 170.61, 157.90, 143.03, 139.88, 137.89, 129.41, 127.77, 121.87, 120.51, 110.27, 47.54, 47.25, 38.89, 38.79, 33.33, 30.40, 29.28, 28.05, 26.51, 23.96, 20.83. ESI–MS (m/z): % theoretical (% experimental): 869.03 (868.62). Elemental analysis: % theoretical (% experimental) for C_44_H_65_N_7_O_11_ (867.60 g.mol^−1^): C = 60.86% (60.26%), H = 7.55% (7.52%), N = 11.30% (11.59%).

*GABA-DFO:* At room temperature, 50 mL of a 40% of dimethylamine (DMA) aqueous solution was slowly added to a mixture of 200 mg (0.23 mmol) of Fmoc-GABA-DFO in 50 mL of ACN. After 1 h, the solvent was eliminated under high pressure, and 9 mL of methanol was added to the solid. The mixture was kept at 4 °C overnight, centrifuged for the separation of white solid, which was washed three times with cold diethyl ether.

Yield: 27%. m.p.: 146.5–149.0. R_f_: 0 (positive to ninhydrin test). ^1^H-NMR (300 MHz, dmso-d_6_): 7.69–7.80 (m, 3H), 3.42–3.50 (m, 10H), 3.01 (q, J = 6.1 Hz, 6H), 2.58 (t, J = 7.2 Hz, 4H), 2.28 (t, J = 7.2 Hz, 4H), 2.08 (q, J = 7.2 Hz, 2H), 1.97 (s, 3H), 1.59 (m, 2H), 1.51 (qn, J = 6.9 Hz, 6H), 1.40 (qn, J = 6.9 Hz, 6H), 1.24 (q, J = 6.9 Hz, 6H). ^13^C NMR (75 MHz, dmso-d_6_): 172.41, 172.27, 171.78, 170.59, 47.52, 47.25, 38.77, 38.88, 33.28, 30.39, 29.27, 28.04, 26.50, 23.95, 20.83. ESI–MS (m/z): % theoretical (% experimental): 646.79 (646.45). Elemental analysis: % theoretical (% experimental) for C_29_H_55_N_7_O_9_.3.65H_2_O (711.54 g.mol^−1^): C = 48.85% (47.52%), H = 8.83% (7.39%), N = 13.78% (12.87%).

### Calcein competition assays

The interaction between the conjugates and iron was assessed by a competitive assay with the fluorescent probe calcein (Espósito et al. 2002). In a black 96-well microplate, 90 µL of 1 µM of ferric calcein complex (CAFe) in HBS (Hepes Buffer Saline: hepes 20 mM, NaCl 150 mM, Chelex 1 g/100 mL; pH 7.4) and 10 µL of 5-ALA-DFO, 5-ALA, GABA-DFO, GABA or DFO (final concentration: 0–8 µM) were added to each well. The fluorescence (λ_exc_/λ_emis_: 485/520 nm) was registered for 90 min at 37 °C. The experiment was performed three times in triplicates.

### Antioxidant activity against iron-dependent oxidation

The ability of the conjugates to inhibit the iron-catalyzed autoxidation of ascorbate, a proxy for the toxicity of free iron in plasma (Espósito et al. 2003), was assessed by transferring 10 µL of 200 µM aqueous ferric nitrilotriacetate (Fe(nta)) and 10 µL of 5-ALA-DFO, 5-ALA, GABA-DFO, GABA or DFO (final concentration: 0–50 µM) in HBS to a black, 96 well microplate. Then, 180 µL of a solution of 40 µM of ascorbate and 50 µM of DHR in HBS were added to the wells, and the fluorescence (λ_exc_/λ_emis_: 485/520 nm) was registered for 60 min at 37 °C, in triplicates. The rates of DHR oxidation (fluorescence units/min) *versus* chelator concentration were obtained for the time range of 15–45 min (Espósito et al. 2003).

### Interaction with Tf

The ability of the conjugates to remove iron from Tf was assayed with the fluorescent probe apo-Tf (Fl-aTf), and the protocol for this experiment was adapted from a published procedure (Breuer and Cabantchik 2001). 180 µL of 2 μM of Fl-aTf in HBS was added to a black 96-well microplate, followed by 10 µL of 40 µM aqueous FAS and 10 µL of conjugates (final concentrations: 0–20 µM). Each new compound was only added after fluorescence stabilization. The experiment was conducted in triplicate and the fluorescence (λ_exc_/λ_emis_: 485/520 nm) was measured for 90 min at 37 °C (Espósito et al. 2002).

### Interaction with human serum albumin (HSA)

#### Fluorescence quenching

In microconcentration tubes, 6.25 µM aqueous solutions of HSA were treated with 5-ALA-DFO or GABA-DFO (final concentrations: 0–6.25 µM) and incubated for 30 min at room temperature. Then, fluorescence emission (FE) spectra of both free and conjugated-treated HSA were obtained in 1 cm quartz cuvette from 320 to 500 nm, with excitation at 290 nm (Espósito et al. 1999).

#### Circular dichroism (CD)

In microconcentration tubes, 0.75 µM aqueous solutions of HSA were treated with 5-ALA-DFO or GABA-DFO (final concentrations: 0–0.75 µM) and incubated for 30 min at room temperature. CD spectra were recorded in the range of 260–200 nm, with a scan speed of 20 nm.min^−1^ in 1 cm quartz cuvettes (Espósito et al. 1999).

### Theoretical calculations

The calculations were performed using the Gaussian 16 program (revision A.03) (Frisch et al. 2016) using computational resources provided the Computer Center of St. Petersburg State University and by Superintendência de Tecnologia da Informação da Universidade de São Paulo. All structures were fully optimized, and the energy minima were checked by analytical calculation of the frequencies of normal vibrations. Geometry optimizations, calculations of the frequencies of normal vibrations, and determinations of thermodynamic potentials were performed at the DFT level using the B3LYP functional (Becke 1993; Lee et al. 1988; Vosko et al. 1980), the 6–311 + + G(2d,2p) basis set was used for all elements (Clark et al. 1983; Frisch et al. 1984). Calculations in water (ε = 78.3553) were performed using the polarized continuum model (PCM) (Tomasi et al. 1999). Chemcraft program (V. 1.8) was used to visualize the optimized geometries and normal vibrations.

### Cell viability

Human colorectal adenocarcinoma cells (Caco-2) were grown in T75 culture flasks with Dulbecco’s modified Eagle’s medium (DMEM) supplemented with 10% fetal bovine serum, 1% L-glutamine, and 1% penicillin/streptomycin at 37 °C in 5% CO_2_. After reaching about 90% of confluence, cells were detached using 0.25% trypsin–EDTA solution, centrifuged at 1500 rpm for 5 min, resuspended in DMEM, and counted using a Neubauer chamber. 5 × 10^3^ cells per 100 μL were transferred to 96-well plates and seeded for 7 days, having the medium exchanged every 2 days. Cells were washed with HBS pH 7.4 and incubated with 0, 25 or 100 µM of 5-ALA-DFO, GABA-DFO, SIH (salicylaldehyde isonicotinoyl hydrazone), DFO, or 5 µM of ferric-8-hydroquinoline (Fe(hq) 1:1) for 24 h. Cells were also treated with 1% DMSO in HBS as a control. After incubation, the cells were washed, treated with 0.05 mg.mL^−1^ MTT solution and incubated for 45 min. The medium was removed, the crystals were dissolved in DMSO, and the absorbance was measured (λ = 570 nm) in a Spectramax i3 (Molecular Devices, USA) microplate reader (Riss et al. 2013). The experiment was performed in triplicate and repeated twice. All the data were normalized in relation to the respective controls.

### Intracellular competition studies with calcein

7.5 × 10^4^ Caco-2 cells per 400 μL were added to 24-well plates and seeded for 7 days. The medium was replaced every 2 days. Then, cells were washed and treated with CAL-AM 1 µM for 20 min at 37 °C. Cells were washed again and exposed to 0 or 5 µM of Fe(hq) for 15 min. After washing, the cells were incubated for 30 min with 0 or 25 µM of 5-ALA-DFO, GABA-DFO, SIH (positive control) or DFO (negative control). Cells were washed and the medium was substituted by indicator-free (colorless) DMEM. Fluorescence microscopy images were analyzed using ImageJ 1.54 g software (NIH, USA). The experiment was made in triplicates and repeated twice. Fluorescence was normalized relative to the blank (cells treated with only CAL-AM) (Cegarra et al. 2022; Goswami et al. 2014).

### Data analysis

Results were expressed as means ± standard deviation (n = 3 replicates). Statistical analyss were performed using GraphPad Prism software (GraphPad Software Inc.). Normal distribution was assessed by the Shapiro-Milk test. One-way analysis of variance (ANOVA) followed by *post-hoc* Tukey’s test was used to distinguish significant differences (p < 0.05).

## Results and discussion

### Synthesis and purification of the conjugates

In this study, we focused on chemically synthesizing, purifying and characterizing conjugates of DFO with non-proteinogenic amino acids reported as permeable to physiological barriers through fast and efficient protocols, avoiding the use or reducing as much as possible the volumes of solvents classified as Hazardous Air Pollutants (HAP) by the Environmental Protection Agency (EPA) (Alfonsi et al. 2008). To that end, the following points were considered: i) classical steps of peptide synthesis in solution would be cheaper and faster; ii) since DFO has a free amine group, and 5-ALA and GABA also have amines and carboxylate groups, the amino acids’ amine groups would be protected and their carboxyl-group would be activated with EDC and HOBt; iii) Boc (tert-butyloxycarbonyl) and Fmoc (9-fluorenylmethyloxycarbonyl) were chosen as protecting groups due to their chemical lability in anhydrous organic media (acidic and basic, respectively), where the amide bond would not be affected or specific undesirable reactions would not take place during deprotection of Boc-5-ALA-DFO and Fmoc-GABA-DFO (Albericio et al. 2009; Okuda-Shinagawa et al. 2017).

Regarding the synthetic route chosen after several analytical-scale attempts, it was based on those widely employed in traditional chemical peptide synthesis (Miranda et al. 1986; Loffredo et al. 2009): amide bond formation (Scheme S1) was carried out in anhydrous ethanol:DMF (1:1, v/v) at apparent pH ~ 8 (measured in pH strips after dropping onto them mixtures of water and the reaction solution, 1:1, v/v) in the presence of EDC, that forms an active ester intermediate that is rapidly attacked by HOBt to form an even more active carbonyl group, which is attacked by the DFO amine to form Boc-5-ALA-DFO or Fmoc-GABA-DFO (Benoiton 2006). Such reaction condition employs less DMF, a toxic solvent included in the list of HAP from EPA (Alfonsi et al. 2008). The Boc-5-ALA-DFO and Fmoc-GABA-DFO formed were soluble in DMSO and insoluble in ACN, acetyl acetate, chloroform, DMF, hexane, methanol, and water.

Boc and Fmoc deprotections employed conditions adapted from published works (Albericio et al. 2009; Ashworth et al. 2010; Benoiton 2006; Miranda et al. 1986). Boc removal (Scheme S2) involved protonation of Boc carbonyl group in TFA/DCM solution with release of tert-butyl cation and formation of CO_2_, and 5-ALA-DFO (Fig. 1d). This conjugate was soluble in DMSO, and ACN and slightly soluble in methanol and water. As to Fmoc removal (Scheme S3), it took place in 20% DMA/ACN solution, a more environmentally friendly alternative for this reaction (Jad et al. 2015). The reaction involved proton abstraction by DMA (pK_a_ 10.73 at 25 °C), Fmoc decomposition and formation of dibenzofulvene, adduct with DMA, and GABA-DFO (Benoiton 2006; Fig. 1e). GABA-DFO was soluble in ACN, methanol and DMSO and slightly soluble in water. Boc-5-ALA-DFO, 5-ALA-DFO, Fmoc-GABA-DFO, and GABA-DFO were characterized by LC-ESI/MS, ^1^H and ^13^C NMR, FTIR and UV–Vis, giving the results expected for their structures (Figures S1–S21, Supporting Information).

It is worth noting that Boc-5-ALA-DFO and 5-ALA-DFO showed peaks in the UV chromatograms and m/z ions in the mass spectra related to the molar masses 774.57 and 674.41, respectively. According to Jaffe & Rajagopalan (1990), 5-ALA moiety has a ketone group highly susceptible to keto-enol tautomerism in the presence of carboxyl groups and in 60%ACN/0.1%TFA/H_2_O solution used for samples preparation and RP-HPLC elution. The MS of the enol compounds are characterized by the presence of peaks related to their loss of water from enolic moiety in ESI/MS fragmentation, [M-H_2_O + H^+^] (m/z 756.57 and 646.44, respectively). Thus, the components with higher RT are in the keto form, and the those with lower RT are in the enolic form (Bechara et al. 2021; Erdtman and Eriksson 2008; Jaffe and Rajagopalan 1990).

5-ALA-DFO and GABA-DFO were both obtained with high degree of purity (> 97% for 5-ALA-DFO – considering peaks related to enolic and keto forms – and > 96% for GABA-DFO), highlighting how significantly the planning of routes and selection of reaction conditions affected the efficiency of our synthetic work.

### Calcein competition and antioxidant assays

Competition studies with the fluorescent probe calcein are based on the strong interaction between iron(III) and calcein (logK_ML_ = 24), which leads to fluorescence quenching. Fluorescence recovery can only be achieved by chelators with similar or higher affinity for iron, therefore making this method a high throughput tool to assess the iron scavenging ability of the new molecules (Breuer et al. 1995).

**Fig. 2:**
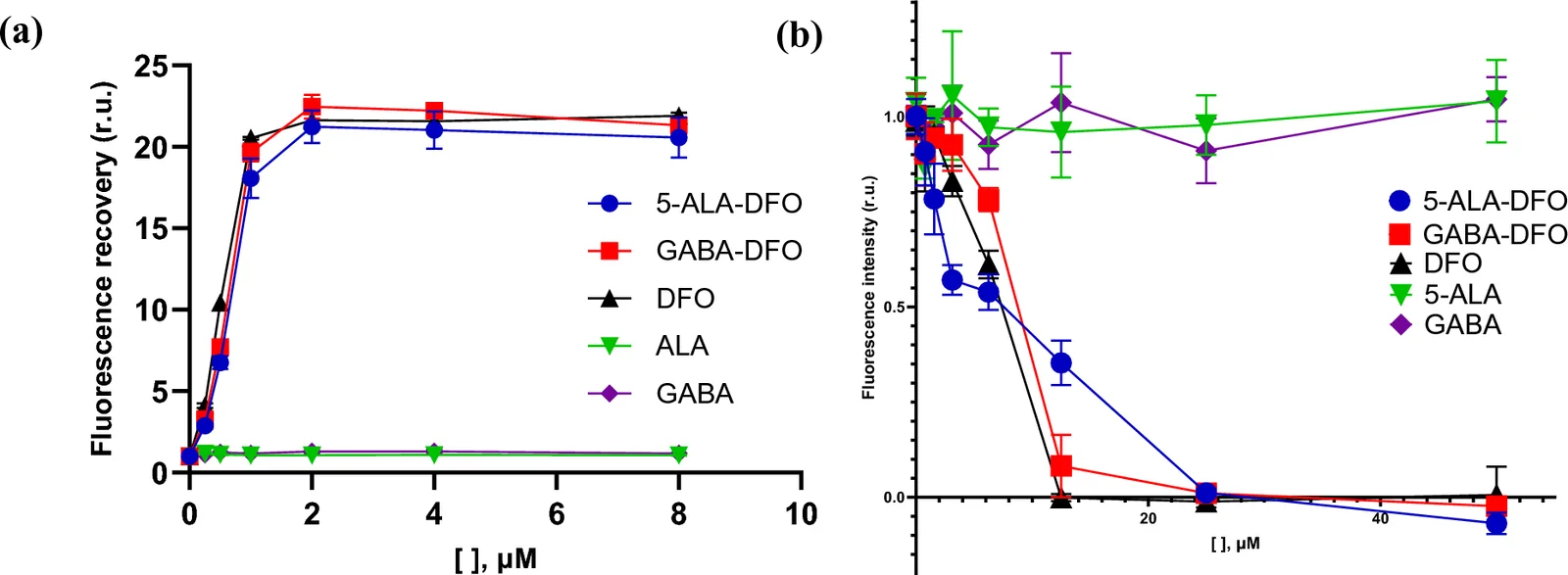
a Calcein fluorescence recovery (%) as a function of chelator concentration; b Antioxidant activity of the proposed chelators against iron-catalyzed ascorbate oxidation, as a function of the rhodamine fluorescence. [CAFe] = 1 µM; [Fe(NTA)] = 10 µM; [ascorbate] = 40 µM; [DHR] = 50 µM. r.u.: relative unit; λexcitation: 485 nm; λemission: 520 nm. r.u.: relative unit

All conjugates and DFO showed a similar profile of fluorescence recovery of calcein within the experimental error (Fig. 2a), indicating that the conjugates conserved the high iron binding of DFO, i.e., conjugation did not affect the metal chelation moiety. On the other hand, the free amino acids 5-ALA and GABA could not compete with calcein as no fluorescence recovery was observed, indicating that chelation is solely dependent on the hydroxamate moiety of DFO.

A similar profile of calcein fluorescence recovery have been reported by our groups for conjugates of DFO with 6-aminoquinoline (DFOQUN), caffeine (DFCAF), and peptides TAT(47–57) (DFO-Suc-TAT(47–57)) and SS02 (DFO-Suc-SS02) (Carvalho et al. 2021; Alta et al. 2014, 2017; Goswami et al. 2014). In all cases, the conjugates bind iron(III) through the hydroxamate moiety of DFO. However, the conjugate of DFO with the lipophilic cation triphenylphosphonium (TPP) exhibited lower affinity to iron than that of DFO but keeping its antioxidant activity in the iron-ascorbate system, which means that a relatively weaker iron-conjugate bond does not necessarily mean the formation of an unstable ferric complex (Alta et al. 2017).

Considering that excess iron can increase the production of ROS via reaction with some species, such as ascorbate, the antioxidant activity of 5-ALA-DFO and GABA-DFO (their ability of trapping Fe(III) in redox-inactive forms) was followed as the decrease in the oxidation rates of the probe DHR. This experiment is based on the oxidation of DHR to rhodamine (RhB) by free radicals such as superoxide or other ROS produced from the reaction between Fe(II) and oxygen. Fe(II) is obtained from reduction of Fe(III) by ascorbate (Esposito et al. 2003; Halliwell and Gutteridge 2007; Herderson and Chappell 1993; Scheme S4). In agreement with the data obtained for iron binding ability, GABA-DFO displayed the same concentration-dependent decrease of DHR oxidation as that of DFO (Fig. 2b). The antioxidant activity of 5-ALA-DFO was somewhat decreased, which may reflect either a lower stability under ascorbate, or a higher reduction potential of its ferric complex in comparison to ferric DFO. In any case, the antioxidant effect of the conjugates was due to iron chelation, since free 5-ALA or GABA were not active in this model.

### Interaction of the conjugates with plasmatic proteins Tf and HSA

Tf is the protein responsible for iron transport and delivery to cells and tissues, maintaining low levels of circulatory free iron (Silva et al. 2021). In general, an elevated Tf saturation is a hallmark of iron overload since the ferric complex in Tf is inert and stable. On average Tf saturation higher than 50% for men or 45% for women are associated with haemochromatosis (Yin et al. 2023). Despite having higher thermodynamic affinity for iron, DFO is not able to scavenge it from Tf (Pollack et al. 1976), due to its inability to access the metal inside the hydrophobic Tf lobes.

**Fig. 3:**
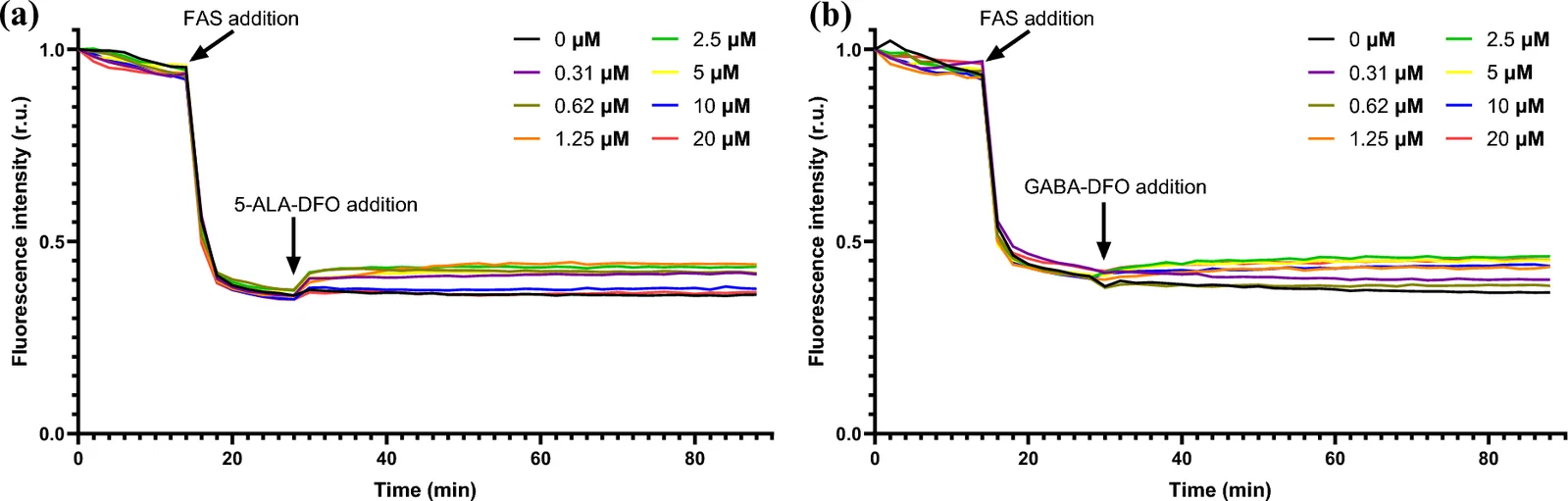
Effects of increasing concentrations of a 5-ALA-DFO, and b GABA-DFO on the fluorescence of fluorescein-apotransferrin (Fℓ-aTf) after addition of iron solution (FAS), respectively. *r.u*.: relative unit

The interplay between iron, the conjugates, and Tf was studied. We wanted to investigate the ability of the conjugates to scavenge transferrin-bound iron. To that end, it was employed an assay with fluorescein-labeled apoTf (Fl-aTf), a fluorescent probe that, similarly to calcein, displays fluorescence quenching upon binding to iron (Breuer and Cabantchik 2001). Here, we chose to work with 50% of Tf saturation and used FAS to load iron to Fl-aTf, since the oxidation of Fe(II) to Fe(III) by oxygen at pH 7.4 is significantly enhanced in the presence of apoTf, leading to the formation of Fe(III)-Tf (Bates et al. 1973). The kinetic profile of fluorescence quenching for 5-ALA-DFO and GABA-DFO (Figs. 3a,b, respectively) showed that the increase in conjugate concentration has no effect on Fl-aTf fluorescence, indicating that both compounds cannot remove iron from Tf within the timeframe of this experiment, as the metal is bound in a protein pocket without access to external chelators. The same behavior was observed for both unmodified DFO and other DFO conjugates (Alta et al. 2017; Goswami et al. 2014; Pachpatil et al. 2026; Pramanik et al. 2019). Unlike 5-ALA-DFO and GABA-DFO, the iron chelator deferiprone (DFP) can remove iron from Tf when its concentration in blood serum is higher than 0.15 mM (Kontoghiorghes 2023).

The interaction between 5-ALA-DFO and GABA-DFO with HSA was also explored. HSA is the most abundant plasma protein, and it has been studied as vehicle for the transport of drugs (Mishra and Heath 2021). The rapid change in protein conformation after interaction with some ligands, and the presence of only one fluorescent tryptophan residue (Trp314) in the protein, make it possible to associate alterations in circular dichroism (CD) spectra and in fluorescence emission (FE) with changes in the secondary structure of HSA.

**Fig. 4:**
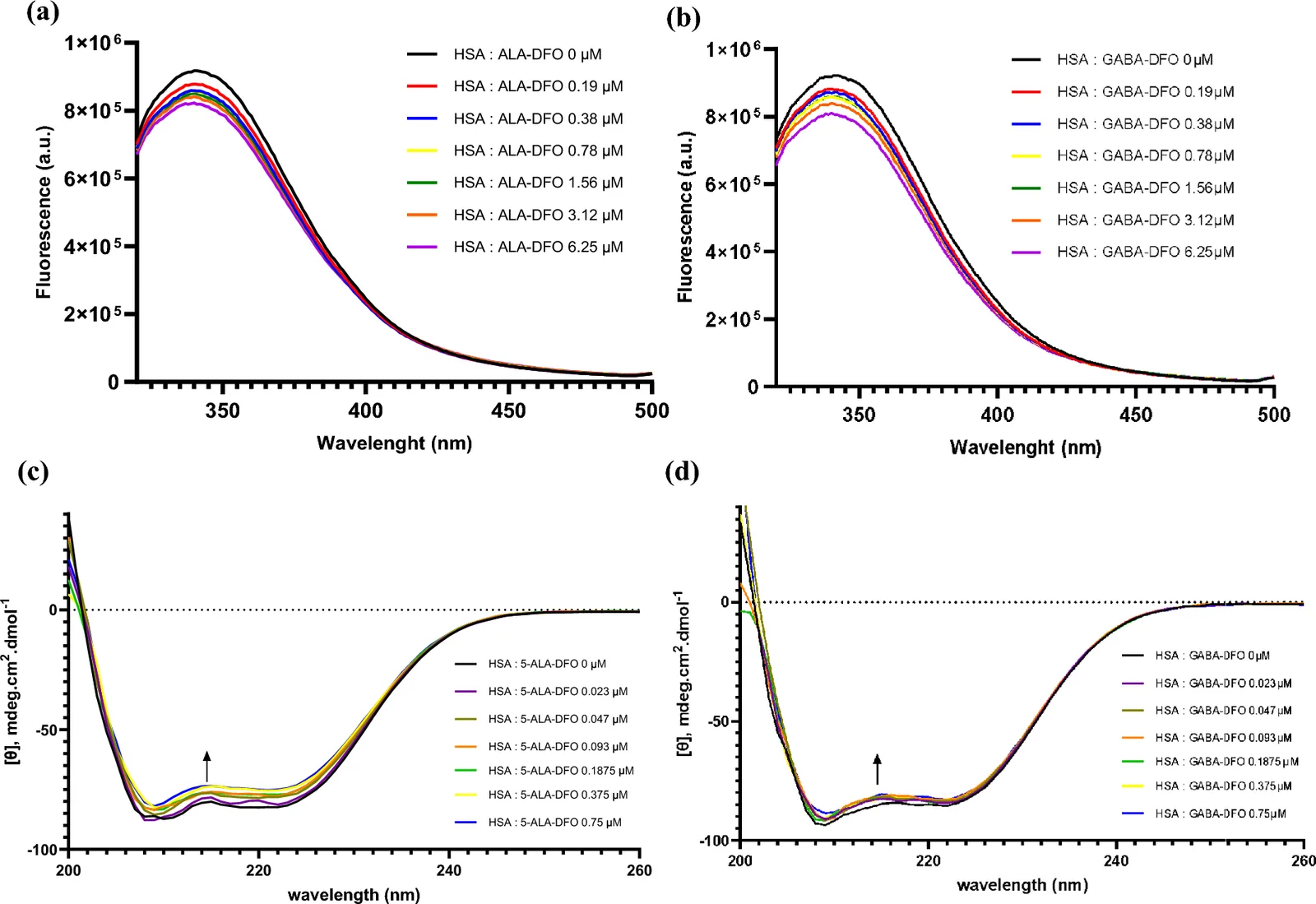
FE of Trp-214 of free HSA (6,25 µM) and in the presence of different concentrations of a 5-ALA-DFO, and b GABA-DFO (λ_exc_ = 290 nm); and CD of free HSA (0.75 µM) and in the presence of different concentrations of c 5-ALA-DFO, and d GABA-DFO. In all experiments, the incubation was performed for 30 min at room temperature. *a.u*.: arbitrary unit

The FE of HSA has a maximum at 340 nm, whose intensity was not reduced upon addition of 5-ALA-DFO and GABA-DFO (Figs. 4a,b, respectively). This result indicated that the structure of the hydrophobic pocket around Trp314 is not significantly disturbed by the conjugate, which would otherwise expose the amino acid to the solvent and thus lead to fluorescence quenching. The CD spectrum of HSA (Figs. 4c, d) displays two negative bands at 209 and 222 nm, related to π → π* and n → π* transitions at the peptide bond on the α-helix structure of the protein. In accordance with CD data, increasing concentrations of 5-ALA-DFO and GABA-DFO did not significantly alter the position and intensities of those transitions, which indicates the stability of the secondary structure of HSA. The minor decrease in the bands of FE and CD of HSA could be related to the dilution of the solutions.

These small changes on FE and CD indicated that, unlike the chelator DFP that interacts strongly with HSA (Dorraji et al. 2014), the conjugates weakly interact with the protein, which may be due to their larger sizes compared to DFP. These data suggest that a possible pharmacological application for the conjugates would not involve transport via HSA.

### Theoretical study of iron binding to the aminoacyl conjugates

The development of new chelators capable of effectively binding iron (or any other metal) in various biological systems is a rather labor-intensive process. On the one hand, it is necessary to develop methods, routes and protocols for synthesizing it; on the other hand, conducting biological tests also requires substantial time. In the framework of this study, a theoretical model based on quantum-chemical calculations was developed to propose how and where iron binds to the new synthetic conjugates. To assess the efficiency of the model, a comparison was made with the available experimental data. This approach simplifies the development of new chelators since it allows a preliminary assessment of the efficiency of a set of various compounds for chelating metals.

On the one hand, the efficiency of the chelation process is determined by the kinetic aspect—how easily the chelator penetrates the cell and how easily it can approach the active site due to its steric structure. On the other hand, the thermodynamic aspect of this interaction determines the energetic efficiency of chelation. DFT calculations allow us to calculate the stability constants of chemical processes based on the calculated enthalpies and entropies of the compounds participating in these reactions. In this study, to calculate the thermodynamics of the chelation process, the structures of all reaction components were calculated—free ligands, ligands coordinated to the iron atom, and fragments of the active site of transferrin, with and without the iron atom as a product of interaction with the ligand. The objects studied were DFO, 5-ALA-DFO, and GABA-DFO, as well as DFO-Suc-SS02 (Fig. S27). The latest ligand was included in the calculations because its high chelating ability was previously shown by our groups (Alta, et al. 2017), and that allows for an opportunity to compare this theoretical approach with existing experimental data.

For the calculation, a functional B3LYP (Becke 1993; Lee et al. 1988; Vosko et al. 1980) was used, which is believed to be suited for the thermodynamic potential calculations for transition metal complexes (Simonova et al. 2024a, 2024b). The coordinates of the atoms for all calculated structures, as well as the structures of the iron complexes and calculated IR spectra of chelators, are given in the Supporting Information (Figures S22–S31, Tables S7–S16). Similarity of the calculated IR spectra with the experimental ones, the absence of negative vibration frequencies and the convergence of the maximum forces as well as RMS forces allowed us to believe that the geometry is optimized correctly and, despite the flat nature of the potential energy surface, the thermodynamic potentials can be used for further calculations.

As a quantitative characteristic of the ligand effectiveness as a chelator, constants were calculated for the following equilibrium:

$${\mathrm{Tf}} - {\text{Fe }} + {\text{ H}}_{{3}} {\mathrm{L}} \rightleftharpoons {\mathrm{TfH}}_{{3}} + {\text{ FeL}}$$

where L = DFO, 5-ALA-DFO, GABA-DFO or DFO-Suc-SS02 (Figure S26), Tf-Fe = fragment of the ferric transferrin active center. The structure of Tf-Fe contains 86 atoms (Figure S27).

Also, the structure of the apo-Tf active center (TfH_3_) was calculated (Figure S27). In these latter cases, the structural parameters of the system did not change; only the position of hydrogen atoms replacing iron atoms was optimized.

The proposed model was intended to answer two questions: 1) which of the considered ligands is a more effective chelator for iron in transferrin; and 2) what are the values of the chelation constants of these processes? Naturally, the proposed model is extremely simple—it does not take into account steric factors, kinetic factors, the influence of the pH values on the protonation of ligands and the pK_a_ values of the transferrin active center. However, the correspondence of this model to the available experimental data will allow it to be used in the future to predict the chelating ability of plenty of ligands.

Iron(III) has a d^5^ configuration and so can have different multiplicity depending on the ligand environment. Also, there are several possibilities to form different geometrical isomers. DFT calculations for the Fe(DFO) complex showed that the fac-isomer with high spin state of the iron atom (multiplicity equal to 6) is more stable, which is in accordance with the literature (Dhungana 2001). This geometry and multiplicity were used in other DFT calculations for complexes between iron and DFO or its conjugates. All structures were calculated in water solution using PCM-model. The coordinates of the atoms for the calculated complexes as well as their structures are given in Supporting Information (SI). The calculating method for the constants of these processes is given in the SI as well. Since only a fragment of the transferrin active center was used in the calculations and due to the simplicity of the proposed model, the values of the calculated equilibrium constants (K_eq_) of the chelating processes themselves were not very reasonable. Nevertheless, these data make it possible to compare the chelating efficiency of the ligands with each other. Thus, it is more useful to use normalized equilibrium constants instead of their calculated values:

$$K_{norm} \left( {{\mathrm{Fe}} - {\mathrm{DFO}}\,{\mathrm{conjugate}}\,{\mathrm{complex}}} \right) = \frac{{{\mathrm{K}}_{{{\mathrm{eq}}}} \left( {{\mathrm{Fe}} - {\mathrm{DFO}}\,{\mathrm{conjugate}}\,{\mathrm{complex}}} \right)}}{{{\mathrm{K}}_{{{\mathrm{eq}}}} \left( {{\mathrm{Fe}} - {\mathrm{DFO}}\,{\mathrm{complex}}} \right)}}$$

where *K*_eq_(*i*) – calculated constant for the iron chelating process by DFO or its conjugates, while *K*_eq_ for the process with DFO is selected as a “standard”. Normalized constants are given in Table 1.

**Table 1:** Normalized calculated equilibrium constants (*K*_norm_) of DFO and its conjugates interactions with Tf active centers with TfH_3_ formation in solution (PCM-model)

Chelator	DFO	5-ALA-DFO	GABA-DFO	DFO-Suc-SS02
*K*_norm_	1	10^64^	10^57^	10^71^

The DFT calculations showed that all conjugates of DFO are more efficient chelators than DFO itself. This result is consistent with the experimental data obtained in our earlier work devoted to MPPs conjugated to DFO as chelators for mitochondrial labile iron (Alta, et al. 2017). In particular, the iron-binding ability of DFO-Suc-SS02 was found to be stronger than DFO. As a result, half maximal inhibitory concentration (IC_50_) for growth rate assays in A2780 cells for this conjugate (ca 42 μM) was significantly lower than for DFO itself (ca 1450 μM). Also, in this work, it was shown that 5-ALA-DFO, GABA-DFO, and DFO-Suc-SS02 are more effective iron chelators compared to DFO. Thus, the calculations performed are in good agreement with the available experimental data.

It should be noted that the proposed calculation model is quite simple, since it uses only a fragment of the active center of transferrin (Figure S27). Naturally, in this case it is impossible to consider different steric factors arising from the interaction of transferrin with both conjugate chelators. To take such factors into account, it is necessary to use a fragment of the active center that is significantly larger in number of atoms in the calculations, which will lead to an increase in the duration of the calculations, negating their main advantage. Thus, it can be concluded that the proposed model is suitable for predicting the overall efficiency of chelators with respect to the metalloenzymes but cannot assess the real values of chelation constants. However, with close chelator efficiency, i.e. with close values of the calculated equilibrium constants, steric factors can play a decisive role in the metal binding process.

### Cell viability and intracellular iron binding assays

Caco-2 cells differentiate and, subsequently, acquire characteristics, such as formation of tight junctions and expression of amino acid and drug transporters (Ohshima et al. 2019). While under differentiation, Caco-2 cells have similarities with and, therefore, been employed as in vitro models to assess the permeability of natural and synthetic compounds through intestinal epithelial cell barrier (Grifoni et al. 2025). For these reasons, the ability of new conjugate chelators to permeate cells and bind intracellular iron was assessed via Caco-2 permeability, being the experiments started 7 days after cell plating, when cell differentiation has already begun (Cai et al. 2014; Cegarra et al. 2022). Besides 5-ALA-DFO and GABA-DFO, the assays were also performed for SIH and DFO (positive and negative controls of cell permeability, respectively).

None of the compounds displayed significant toxicity to Caco-2 cells (Figure S28, SI), since all treatments led to > 90% survival after 24 h exposure*.* In HeLa cells, other DFO conjugates such as DFOQUN and DFOANI displayed higher toxicity than unmodified DFO, suggesting that the compound conjugated to DFO can influence the cytotoxicity of the chelator (Carvalho 2019).

**Fig. 5:**
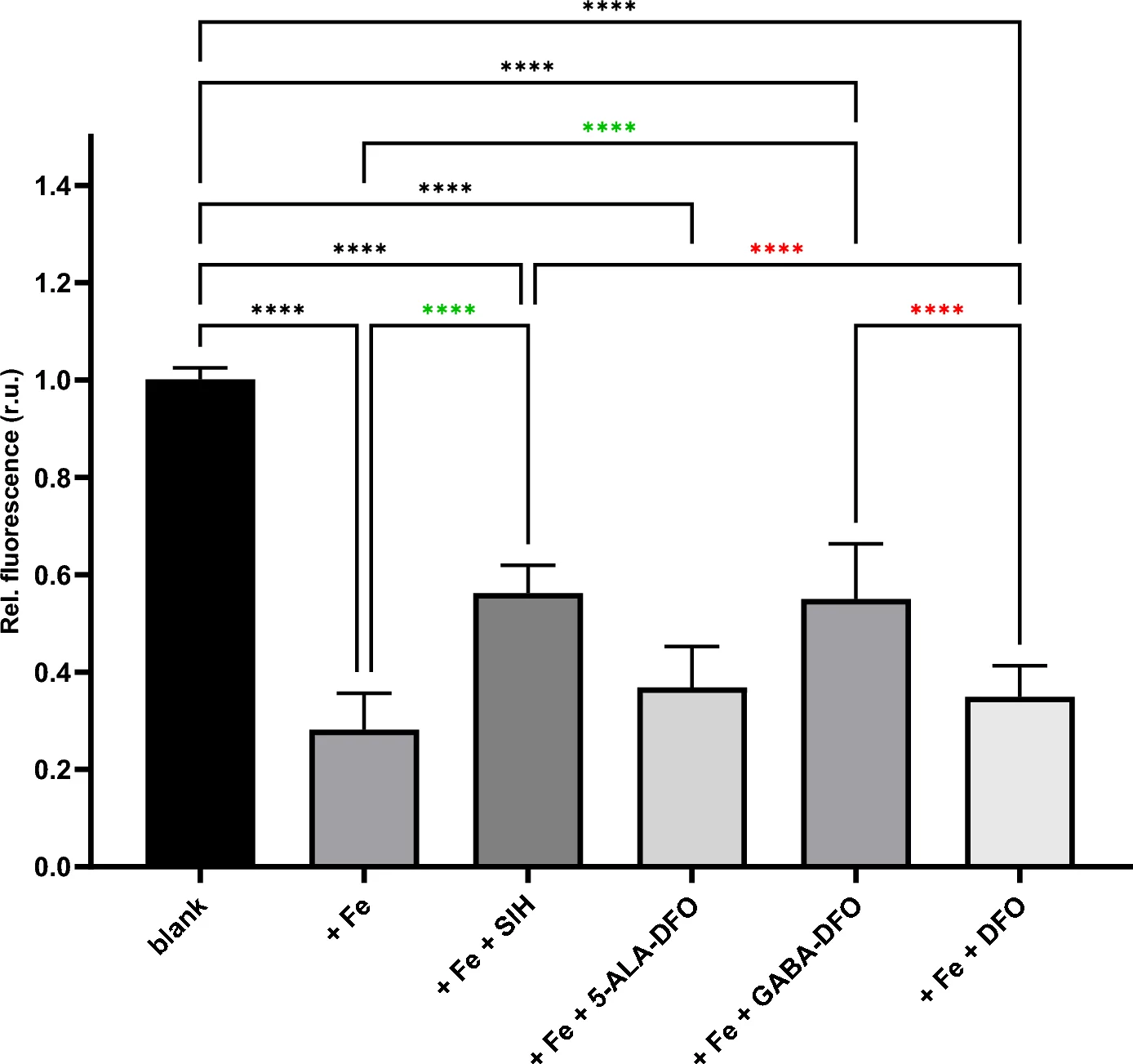
Fluorescence Recovery (%) of calcein after exposure to 1 µM of CAL-AM, 0 or 5 µM of Fe(hq) and with 0 or 25 µM of SIH, 5-ALA-DFO, GABA-DFO or DFO in Caco-2 cells. Experimental data are expressed as mean values ± SD from n = 3 independent assays performed in triplicate. Statistical analysis was performed through unidimensional ANOVA, followed by post hoc Tukey test (**** means p < 0,0001, and *** p < 0,001, being black “in relation to blank”, green “in relation to + Fe”, and red “in relation to + Fe + DFO”, negative control)

CAL-AM, the nonfluorescent, cell permeable form of calcein (Cegarra et al. 2022), is rapidly converted by cytosolic esterases into fluorescent calcein, which binds intracellular iron, forming the quenched complex CAFe. In our experiment, the incubation with the lipophilic complex Fe(hq) induced a fast and efficient absorption of iron into the cells and a decrease of fluorescence was rapidly observed (Fig. 5) (Epsztejn et al. 1997, Leanderson and Tagesson 1996).

**Fig. 6:**
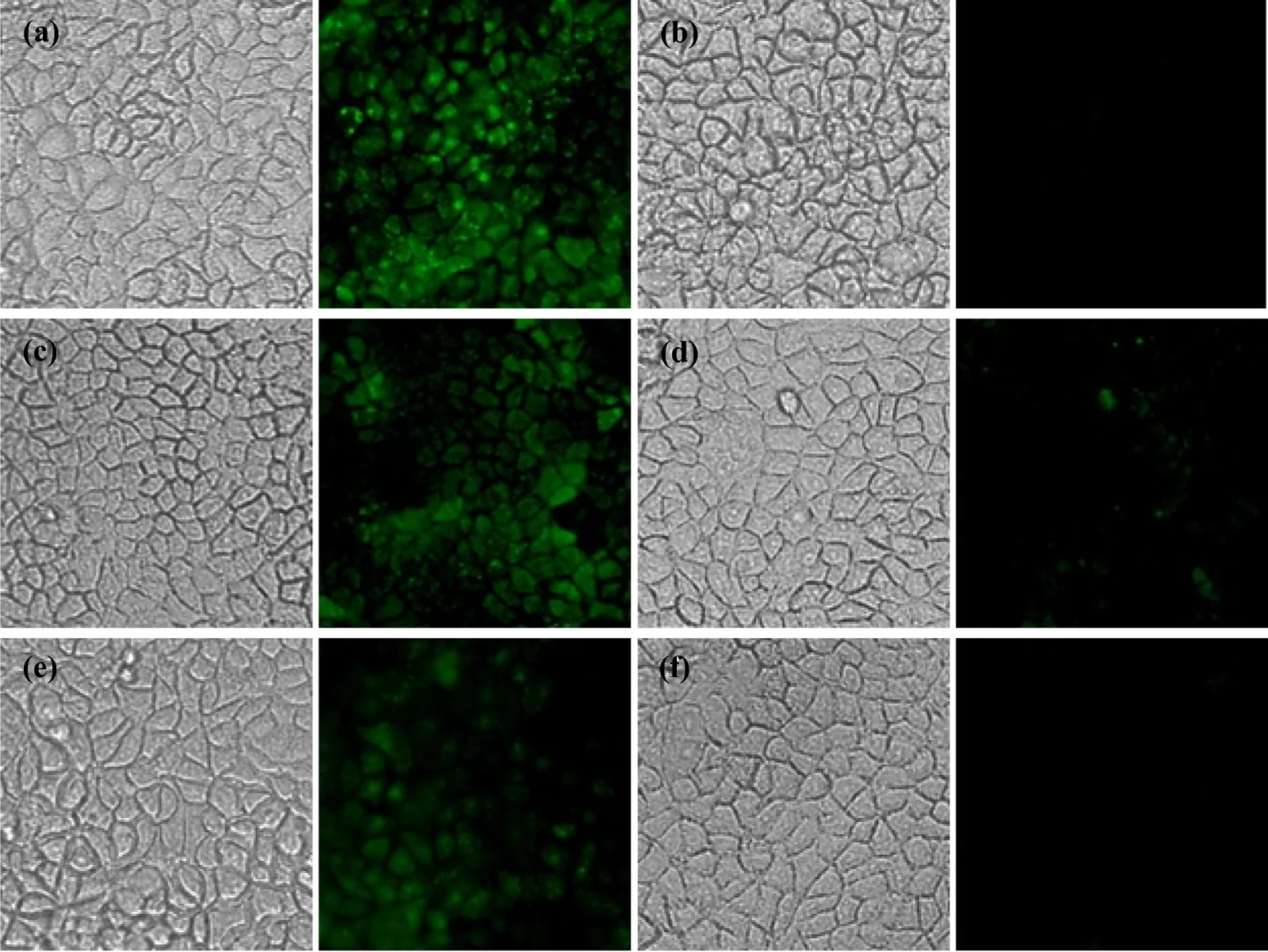
Representative brightfield and fluorescence microscopy images of Caco-2 cells after exposure to (a) CAL-AM; (b) CAL-AM and Fe(hq); and CAL-AM, Fe(hq) and 25 µM of (c) SIH, (d) 5-ALA-DFO, (e) GABA-DFO or (f) DFO

Therefore, if the conjugates 5-ALA-DFO or GABA-DFO are permeable, an increase in fluorescence would be observed. Unmodified DFO was the negative control, since it shows low cell permeability (Farr and Xiong 2021), and SIH, a lipophilic tridentate iron chelator, was the positive control (Epsztejn et al. 1997). After SIH treatment, a significant (although not complete) fluorescence recovery was observed (Figs. 5 and Fig. 6). Interestingly, similar results were observed for GABA-DFO, but not for 5-ALA-DFO.

Altogether, such experiments suggest that, unlike GABA, the conjugation to DFO may have affected 5-ALA ability to transverse cell membranes. Thus, GABA-DFO could be listed along other cell permeable DFO conjugates (Alta et al. 2014; Carvalho et al. 2021; Goswami et al. 2014). Furthermore, in combination with the GABA conjugates with cytoplasmic transduction peptide (Li et al. 2017), which exhibited blood–brain barrier permeability, and with E_2_-GABA and AA-GABA, which showed favorable interactions with GABA_A_-receptor and cerebrovascular effects in rats with ischemic brain injury (Gan’shina et al. 2024), the findings for GABA-DFO further indicate that the GABA moiety in this conjugate enhances the cellular penetration of DFO.

## Conclusions

The new conjugates of DFO with 5-ALA and GABA were designed, synthesized, purified, and characterized. 5-ALA-DFO and GABA-DFO bind iron with affinity comparable to that of DFO, both showed antioxidant activity in the iron/ascorbate system, were not able to remove iron from transferrin, and weakly interacted with human serum albumin. Furthermore, the computational simulation results were consistent with the published experimental data, confirming that this theoretical quantum-chemical model can be a valuable tool for the design of new iron chelators. Both conjugates were not harmful to Caco-2 cells. Notably, GABA-DFO showed enhanced cellular permeability relative to DFO and could bind cytosolic iron within Caco-2, suggesting that the conjugation strategy may have improved DFO cell permeability. Considering the promising characteristics of GABA-DFO as an iron chelator, this work opens up the possibility for the investigation of its neuroprotective effects using more complex biological models.

## Supplementary Information

Below is the link to the electronic supplementary material.

Supplementary file1 (DOCX 4949 KB)

## Data Availability

No datasets were generated or analysed during the current study.
