# Supplementary material for: Conjugates of desferrioxamine with 5-aminolevulinic or γ-aminobutyric acid: synthesis, theoretical studies, iron-chelating ability, and penetration in human Caco-2 cells
Source: Biometals. 2026 Jun 24;39(5):1921–37. doi: 10.1007/s10534-026-00841-7 (PMC13518394; doi:10.1007/s10534-026-00841-7)
Supplement: Supplementary file 1 [file 10534_2026_841_MOESM1_ESM.docx]

**Conjugates of desferrioxamine with 5-aminolevulinic or γ-aminobutyric acid: synthesis, theoretical studies, iron-chelating ability, and penetration in human Caco-2 cells**

Ernani Lacerda, Vasilii D. Khripun, M. Teresa Machini, Breno Pannia Esposito

**Supporting Information**

**Scheme S1.** Synthetic route for the formation of the active ester and subsequent formation of the amide bond, yielding Boc-5-ALA-DFO or Fmoc-GABA-DFO.

**Scheme S2.** Proposed mechanism for Boc deprotection in 50% TFA/DCM to obtain 5-ALA-DFO (H_3_N^+^-5-ALA-DFO).

**Scheme S3.** Proposed mechanism of Fmoc removal in DMA solution to obtain GABA-DFO.

**Scheme S4.** Chemical equations for antioxidant assays in dihydrorhodamine-ascorbate system (Esposito et al., 2003; Halliwell and Gutteridge 2015).

a) Fe(III) + ascorbate ⇌ Fe(II) + ascorbyl radical

b) Fe(II) + O_2_ ⇌ Fe(III) + O_2_^•^

c) DHR + O_2_^•^ ⇌ RhB + byproducts

**Figure S1.** **(a)** UV-vis chromatogram of Boc-5-ALA-DFO and mass spectrum for components eluted at RT **(b)** 11.3-12.3 min, **(c)** and **(d)** 17.3-17.8 min, and **(e)** 17.9-18.2 min.

**(a)**

**(b)**

**(e)**

**(d)**

**(c)**

**Figure S2.** **(a)** UV-vis chromatogram of 5-ALA-DFO and mass spectrum for components eluted at RT **(b)** and **(c)** 11.8-12.1 min, and **(d)** 12.3-12.7 min.

**(a)**

**(b)**

**(c)**

**(d)**

**Figure S3. (a)** UV-vis chromatogram of Fmoc-GABA-DFO and **(b)** mass spectrum for component eluted at RT 24.2-24.6 min.

**(a)**

**(b)**

**Figure S4. (a)** UV-vis chromatogram of GABA-DFO and **(b)** mass spectrum for component eluted at RT 13.1-13.5 min.

**(a)**

**(b)**

**Figure S5.** ^1^H NMR spectrum (in dmso-d_6_) of Boc-5-ALA-DFO.

**Figure S6.** Structural formula of Boc-5-ALA-DFO containing the identification of each hydrogen (a-p, in **green**) and carbon atoms (1-19, in **red**).

**Figure S7.** ^13^C NMR spectrum (in dmso-d_6_) of Boc-5-ALA-DFO.

**Figure S8.** ^1^H NMR spectrum (in dmso-d_6_) of 5-ALA-DFO.

**Figure S9.** Structural formula of 5-ALA-DFO containing the identification of each hydrogen (a-o, in **green**) and carbon atoms (1-16, in **red**).

**Figure S10.** Keto-enolic equilibrium of 5-ALA-DFO (corresponding numbers to each hydrogen and carbon atoms are highlighted in green and red, respectively).

**Figure S11.** ^13^C NMR spectrum (in dmso-d_6_) of 5-ALA-DFO.

**Table S2.** ^1^H and ^13^C NMR data and peak assignments for 5-ALA-DFO. For ^1^H NMR, it is also shown the multiplicity (Mult) and number of hydrogen atoms (N. of H) related to each peak.

| ^1^H NMR | | | | | | | | ^13^C NMR | | | |
| --- | --- | --- | --- | --- | --- | --- | --- | --- | --- | --- | --- |
| H | **δ (ppm)** | **Mult.*** | **N. of H** | **H** | **δ (ppm)** | **Mult.*** | **N. of H** | **C** | **δ (ppm)** | **C** | **δ (ppm)** |
| a | 1.96 | s | 3 | **j** | 2.57 | t | 4 | **1** | 28.02 | **11** | 172.42 |
| b | 9.64 | m | 3 | **k** | 7.90 | t | 1 | **2** | 171.77 | **12** | 170.99 |
| c | 3.45 | t | 6 | **l** | 2.38 | t | 2 | **3** | 47.55 | **13** | 35.08 |
| d | 1.49 | m | 6 | **m** | 2.26 | m | 2 (+4) | **4** | 26.51 | **14** | 30.35 |
| e | 1.21 | m | 6 | **m*** | 2.68 | t | 1 | **5** | 20.83 | **14*** | 30.86 |
| f | 1.38 | m | 6 | **n** | 3.95 | s | 1 | **6** | 23.96 | **15** | 203.89 |
| g | 2.99 | m | 6 | **n*** | 6.60 | s | 0,5 | **7** | 47.29 | **15*** | 173.87 |
| h | 7.79 | t | 2 | **o** | 7.99 | s | 3 | **8** | 170.60 | **16** | 44.57 |
| i | 2.26 | m | 4 (+2) |  |  |  |  | **9** | 29.29 | **16*** | 88.76 |
| *s: singlet; d: dublet; t: triplet; q: quintet; m: multiplet; *related to the keto form of the compound | | | | | | | | **10** | 38.90 |  |  |

**Figure S12.** ^1^H NMR spectrum (in dmso-d_6_) of Fmoc-GABA-DFO.

**Figure S13.** Structural formula of Fmoc-GABA-DFO containing the identification of each hydrogen (a-u, in **green**) and carbon atoms (1-24, in **red**).

**Figure S14.** ^13^C NMR spectrum (in dmso-d_6_) of Fmoc-GABA-DFO.

**Table S3.** ^1^H and ^13^C NMR data and peak assignments for Fmoc-GABA-DFO. For ^1^H NMR, it is also shown the multiplicity (Mult) and number of hydrogen atoms (N. of H) related to each peak.

| ^1^H NMR | | | | | | | | ^13^C NMR | | | |
| --- | --- | --- | --- | --- | --- | --- | --- | --- | --- | --- | --- |
| H | **δ (ppm)** | **Mult.*** | **N. of H** | **H** | **δ (ppm)** | **Mult.*** | **N. of H** | **C** | **δ (ppm)** | **C** | **δ (ppm)** |
| a | 1.98 | s | 3 | **l** | 2.06 | t | 2 | **1** | 28.05 | **13** | 33.33 |
| b | 9.65 | s | - | **m** | 1.62 | m | 2 | **2** | 171.80 | **14** | 30.40 |
| c | 3.47 | m | 6 (+2) | **n** | 3.44 | m | 2 (+6) | **3** | 47.25 | **15** | 38.79 |
| d | 1.51 | m | 6 | **o** | 9.65 | s | - | **4** | 26.51 | **16** | 157.90 |
| e | 1.24 | m | 6 | **p** | 4.31 | m | 1 | **5** | 20.83 | **17** | 120.51 |
| f | 1.40 | q | 6 | **q** | 4.24 | m | 1 | **6** | 23.96 | **18** | 110.27 |
| g | 3.02 | m | 6 | **r’** | 7.82 | m | 1 (+2) | **7** | 47.54 | **19** | 127.77 |
| h | 7.82 | m | 2 (+1) | **r’’** | 7.70 | d | 1 | **8** | 170.61 | **20** | 129.41 |
| i | 2.28 | t | 4 | **s** | 7.36 | m | 2 | **9** | 29.28 | **21** | 137.89 |
| j | 2.59 | t | 4 | **t** | 7.43 | m | 2 | **10** | 38.89 | **22** | 121.87 |
| k | 6.30 | s | 1 | **u** | 7.91 | d | 2 | **11** | 172.43 | **23** | 143.03 |
| *s: singlet; d: dublet; t: triplet; q: quintet; m: multiplet | | | | | | | | **12** | 172.14 | **24** | 139.88 |

**Figure S15.** ^1^H NMR spectrum (in dmso-d_6_) of GABA-DFO.

**Figure S16.** Structural formula of GABA-DFO containing the identification of each hydrogen (a-o, in **green**) and carbon atoms (1-15, in **red**).

**Figure S17.** ^13^C NMR spectrum (in dmso-d_6_) of GABA-DFO.

**Table S4.** ^1^H and ^13^C NMR data and peak assignments for GABA-DFO. For ^1^H NMR, it is also shown the multiplicity (Mult) and number of hydrogen atoms (N. of H) related to each peak.

| ^1^H NMR | | | | | | | | ^13^C NMR | | | |
| --- | --- | --- | --- | --- | --- | --- | --- | --- | --- | --- | --- |
| H | **δ (ppm)** | **Mult.*** | **N. of H** | **H** | **δ (ppm)** | **Mult.*** | **N. of H** | **C** | **δ (ppm)** | **C** | **δ (ppm)** |
| a | 1.97 | s | 3 | **i** | 2.29 | t | 4 | **1** | 28.04 | **9** | 29.27 |
| b | - | - | - | **j** | 2.58 | t | 4 | **2** | 171.78 | **10** | 38.88 |
| c | 3.46 | t | 6 (+2+2) | **k** | 7.74 | m | 1 (+2) | **3** | 47.25 | **11** | 172.41 |
| d | 1.51 | q | 6 | **l** | 2.09 | m | 2 | **4** | 26.50 | **12** | 172.27 |
| e | 1.24 | m | 6 | **m** | 1.59 | m | 2 | **5** | 20.83 | **13** | 33.28 |
| f | 1.40 | q | 6 | **n** | 3.46 | t | 2 (+6+2) | **6** | 23.95 | **14** | 30.39 |
| g | 3.01 | m | 6 | **o** | 3.54 | m | 2 (+6+2) | **7** | 47.52 | **15** | 38.77 |
| h | 7.74 | m | 2 (+1) |  |  |  |  | **8** | 170.59 | **-** | - |
| *s: singlet; d: dublet; t: triplet; q: quintet; m: multiplet | | | | | | | | | | | |

**Figure S18.** FTIR spectra of DFO, Boc-5-ALA, Boc-5-ALA-DFO, and 5-ALA-DFO.

**Table S5.** FTIR data of DFO, Boc-5-ALA, Boc-5-ALA-DFO, and 5-ALA-DFO.

| **Attribution** | **DFO** | **Boc-5-ALA** | **Boc-5-ALA-DFO** | **5-ALA-DFO** |
| --- | --- | --- | --- | --- |
| **νN-H** | 3300 cm^-1^ |  | 3302 cm^-1^ | 3298 cm^-1^ |
| **νO-H** | 3091 cm^-1^ | 3378 cm^-1^ | 3098 cm^-1^ | 3098 cm^-1^ |
| **ν_s_C-H (CH_2_)** | 2926 cm^-1^ | 2986, 2972, 2918, 2876 cm^-1^ | 2959, 2928, 2894 cm^-1^ | 2927, 2890 cm^-1^ |
| **ν_s_C-H (CH_3_)** | 2855 cm^-1^ |  | 2855 cm^-1^ | 2851 cm^-1^ |
| **νC=O** | 1620 cm^-1^ | νC=O: 1726cm^-1^  νC=O^...^H (dimer): 1682 cm^-1^ | 1616 cm^-1^ | 1615 cm^-1^ |
| **δC-H (CH_2_), νC-N or δC-N-H** | 1560 cm^-1^ |  | 1561 cm^-1^ | 1559 cm^-1^ |
| **ν_ass_COO** |  | 1502 cm^-1^ | - | - |
| **ν_s_COO** |  | 1382 cm^-1^ | - | - |
| **νC-O** |  | 1098 cm^-1^ | - | - |
| **νC-N + δN-H** | 1153 cm^-1^ | 1236, 1165 cm^-1^ | 1251, 1161 cm^-1^ | 1252, 1162 cm^-1^ |

**Figure S19.** FTIR spectra of DFO, Fmoc-GABA, Fmoc-GABA-DFO, and GABA-DFO.

**Table S6.** FTIR data of DFO, Boc-GABA, Fmoc-GABA-DFO, and GABA-DFO.

| **Attribution** | **DFO** | **Fmoc-GABA** | **Fmoc-GABA-DFO** | **GABA-DFO** |
| --- | --- | --- | --- | --- |
| **νN-H** | 3306 cm^-1^ | 3330 cm^-1^ | 3299 cm^-1^ | 3300 cm^-1^ |
| **νO-H** | 3098 cm^-1^ | 2530-3065 cm^-1^ | 3094 cm^-1^ | 3097 cm^-1^ |
| **ν_s_C-H (arom.)** |  | 3006-3068 cm^-1^ | 3013-3066 cm^-1^ | - |
| **ν_s_C-H (CH_2_)** | 2928 cm^-1^ | 2937, 2883 cm^-1^ | 2927, 2890 cm^-1^ | 2927, 2890 cm^-1^ |
| **ν_s_C-H (CH_3_)** | 2855 cm^-1^ |  | 2851 cm^-1^ | 2851 cm^-1^ |
| **νC=O** | 1618 cm^-1^ | 1690 cm^-1^ | 1615 cm^-1^ | 1615 cm^-1^ |
| **νC=C, νC-N or δC-N-H** | 1561 cm^-1^ |  | 1560 cm^-1^ | 1559 cm^-1^ |
| **ν_ass_COO** |  | 1548 cm^-1^ | - | - |
| **ν_s_COO** |  | 1414 cm^-1^ | - | - |
| **νC-O** |  | 1321 cm^-1^ | - | - |
| **νC-N + δN-H** | 1159 cm^-1^ | 1258, 1138 cm^-1^ | 1252, 1152 cm^-1^ | 1252, 1162 cm^-1^ |

**Figure S20**. Electronic spectra in the range of 250 e 500 nm **(a)** of 3 mM solutions of DFO (in water), 5-ALA (in water), Boc-5-ALA (in water), Boc-5-ALA-DFO (in DMSO), and 5-ALA-DFO (in DMSO); and **(b)** of 0.75 mM solutions in DMSO of DFO, Fmoc-GABA, Fmoc-GABA-DFO, and GABA-DFO (the enlarged spectra is on the right)

**(a)**

**(b)**

**Figure S21.** Electronic spectra in the range of 250 e 400 nm 0.5 mM solutions of FAS (in water and in 2.5% of DMSO), Fe(DFO) (in water), Fe(GABA-DFO) (in 2,5% of DMSO), and Fe(5-ALA-DFO) (in 2,5% of DMSO).

**Table S7.** Coordinates of atoms for DFO calculated structure

N	18.040372000	-1.792491000	-1.106119000

C	16.971515000	-1.289844000	-0.230738000

C	15.551332000	-1.336823000	-0.813165000

C	14.484467000	-0.797766000	0.147652000

C	13.067356000	-0.844263000	-0.436468000

C	12.023102000	-0.293543000	0.537562000

N	10.671622000	-0.317000000	-0.015241000

C	9.567220000	-0.722409000	0.693286000

O	10.466821000	0.618304000	-1.047996000

C	8.208448000	-0.399177000	0.095078000

O	9.688469000	-1.343248000	1.751003000

C	7.724126000	1.002047000	0.506203000

C	6.388967000	1.341501000	-0.153579000

N	5.534099000	2.073222000	0.603833000

C	4.224359000	2.503079000	0.126962000

O	6.132162000	1.000172000	-1.312011000

C	3.099303000	1.518610000	0.472914000

C	1.733631000	1.989825000	-0.039884000

C	0.602837000	1.004598000	0.279306000

C	-0.746318000	1.483042000	-0.260778000

N	-1.826193000	0.537178000	0.012070000

O	-1.731552000	-0.651559000	-0.739870000

C	-3.092301000	0.931226000	0.377070000

C	-4.180141000	-0.124340000	0.258234000

O	-3.299248000	2.068006000	0.803849000

C	-5.485968000	0.318313000	0.915161000

C	-6.662311000	-0.544340000	0.467585000

N	-7.610001000	-0.770703000	1.412821000

O	-6.749030000	-0.988113000	-0.682015000

C	-8.858340000	-1.469666000	1.130521000

C	-9.995510000	-0.533822000	0.699187000

C	-11.298082000	-1.289184000	0.411367000

C	-12.438682000	-0.365204000	-0.031898000

C	-13.725032000	-1.138608000	-0.330077000

N	-14.811430000	-0.270002000	-0.773641000

C	-16.112408000	-0.410308000	-0.351643000

C	-17.158336000	0.334197000	-1.154723000

O	-16.383527000	-1.075404000	0.648792000

O	-14.620368000	0.258804000	-2.065412000

H	17.821824000	-2.751954000	-1.372532000

H	18.022808000	-1.267270000	-1.979706000

H	17.000959000	-1.868124000	0.701951000

H	17.218515000	-0.255363000	0.041417000

H	15.529524000	-0.758821000	-1.748012000

H	15.311244000	-2.374572000	-1.084901000

H	14.511259000	-1.374782000	1.082525000

H	14.731070000	0.237917000	0.420243000

H	13.037363000	-0.262432000	-1.366375000

H	12.803137000	-1.876781000	-0.697637000

H	11.980862000	-0.892942000	1.448497000

H	12.268351000	0.739406000	0.818501000

H	10.575327000	0.113612000	-1.870736000

H	8.222629000	-0.457854000	-0.993783000

H	7.519538000	-1.157451000	0.475957000

H	7.656067000	1.080440000	1.595906000

H	8.451447000	1.750350000	0.170493000

H	5.780997000	2.267781000	1.563385000

H	4.012753000	3.487790000	0.556784000

H	4.298377000	2.620001000	-0.957305000

H	3.343894000	0.541526000	0.038365000

H	3.060913000	1.380640000	1.561287000

H	1.497410000	2.970425000	0.395932000

H	1.786703000	2.141775000	-1.126814000

H	0.839429000	0.026064000	-0.156660000

H	0.524682000	0.860726000	1.364247000

H	-1.047646000	2.421537000	0.207607000

H	-0.687663000	1.646217000	-1.345462000

H	-1.342998000	-1.301523000	-0.131852000

H	-4.358686000	-0.325422000	-0.803507000

H	-3.826547000	-1.069625000	0.681148000

H	-5.395365000	0.320948000	2.005769000

H	-5.702906000	1.351812000	0.621303000

H	-7.503553000	-0.332481000	2.316487000

H	-8.649135000	-2.194665000	0.339670000

H	-9.145516000	-2.027177000	2.028281000

H	-10.165580000	0.214065000	1.484632000

H	-9.677048000	0.014511000	-0.196048000

H	-11.116862000	-2.042797000	-0.367429000

H	-11.605568000	-1.843256000	1.309064000

H	-12.641639000	0.380568000	0.746948000

H	-12.132860000	0.186129000	-0.929846000

H	-13.544112000	-1.898038000	-1.102751000

H	-14.096219000	-1.644117000	0.563127000

H	-16.905903000	1.392460000	-1.260080000

H	-17.236062000	-0.081753000	-2.163188000

H	-18.114596000	0.232436000	-0.641899000

H	-14.289122000	1.160080000	-1.920567000

**Table S8.** Coordinates of atoms for 5-ALA-DFO calculated structure

N	-7.648275000	7.056106000	0.835253000

C	-11.344774000	3.122370000	-0.159764000

N	-11.177746000	2.085289000	-1.018866000

C	-10.840317000	0.736564000	-0.580410000

C	-9.330568000	0.461349000	-0.575570000

C	-8.996401000	-0.962431000	-0.116170000

C	-7.490351000	-1.250573000	-0.110537000

C	-7.178000000	-2.672507000	0.361201000

N	-5.745098000	-2.954889000	0.379738000

C	-5.205212000	-4.141106000	-0.055943000

O	-5.051410000	-2.284151000	1.406313000

C	-3.762111000	-4.405725000	0.344026000

O	-5.861309000	-4.911447000	-0.758831000

C	-3.170109000	-5.597198000	-0.406321000

C	-1.864727000	-6.069670000	0.226824000

N	-0.933460000	-6.532793000	-0.645421000

C	0.363913000	-7.053443000	-0.232375000

O	-1.693496000	-6.065779000	1.450215000

C	1.501609000	-6.035764000	-0.388527000

C	2.861382000	-6.612876000	0.022195000

C	4.009849000	-5.610025000	-0.138156000

C	5.361169000	-6.208447000	0.269321000

N	6.487110000	-5.300261000	0.070341000

O	6.747199000	-4.993726000	-1.278844000

C	6.890654000	-4.387699000	1.010904000

C	7.895072000	-3.336541000	0.572035000

O	6.462602000	-4.446218000	2.165911000

C	7.188724000	-2.062875000	0.079417000

C	8.175657000	-0.912284000	-0.112469000

N	7.876413000	-0.058091000	-1.123829000

O	9.164240000	-0.771685000	0.613005000

C	8.636090000	1.158898000	-1.386296000

C	8.059975000	2.398012000	-0.688151000

C	8.863366000	3.667375000	-0.993917000

C	8.301635000	4.912176000	-0.296750000

C	9.108545000	6.170362000	-0.624771000

N	8.573656000	7.364043000	0.023516000

C	9.363618000	8.325521000	0.608085000

C	8.687395000	9.642709000	0.925832000

O	10.540555000	8.092216000	0.885712000

O	7.344734000	7.796209000	-0.512736000

O	-11.279304000	2.992349000	1.066551000

C	-11.689971000	4.461205000	-0.809888000

C	-11.193960000	5.643669000	0.018156000

C	-9.681603000	5.779033000	0.033321000

O	-8.952070000	4.999546000	-0.560075000

C	-9.100889000	6.947024000	0.833036000

H	-7.261959000	6.177819000	1.177916000

H	-7.329898000	7.111141000	-0.130980000

H	-11.169693000	2.278196000	-2.010067000

H	-11.247739000	0.614553000	0.426266000

H	-11.352403000	0.025047000	-1.237345000

H	-8.930015000	0.629016000	-1.583963000

H	-8.843064000	1.191049000	0.082796000

H	-9.402088000	-1.124215000	0.892046000

H	-9.502740000	-1.685658000	-0.770409000

H	-7.078074000	-1.109185000	-1.117507000

H	-6.983779000	-0.531869000	0.545726000

H	-7.581619000	-2.839443000	1.368825000

H	-7.617994000	-3.413691000	-0.308210000

H	-4.654939000	-1.508985000	0.976054000

H	-3.163160000	-3.503739000	0.186187000

H	-3.724443000	-4.599714000	1.421319000

H	-3.878413000	-6.432778000	-0.369094000

H	-3.029640000	-5.360265000	-1.465445000

H	-1.124690000	-6.465232000	-1.634685000

H	0.266998000	-7.354731000	0.813355000

H	0.579312000	-7.951820000	-0.821974000

H	1.544206000	-5.698368000	-1.432570000

H	1.270513000	-5.151823000	0.219002000

H	2.813406000	-6.950946000	1.066588000

H	3.073268000	-7.507307000	-0.580200000

H	4.069592000	-5.276227000	-1.180623000

H	3.818164000	-4.720262000	0.474294000

H	5.367378000	-6.469611000	1.329328000

H	5.569852000	-7.117947000	-0.304613000

H	7.411237000	-5.646257000	-1.558005000

H	8.495689000	-3.087347000	1.448616000

H	8.555990000	-3.720248000	-0.207521000

H	6.636724000	-2.261250000	-0.843976000

H	6.456651000	-1.740906000	0.831236000

H	7.024341000	-0.208102000	-1.645100000

H	9.658830000	0.977076000	-1.046689000

H	8.665553000	1.314329000	-2.470075000

H	7.016505000	2.536255000	-1.000201000

H	8.044760000	2.216768000	0.393780000

H	9.908674000	3.519775000	-0.689447000

H	8.880603000	3.834747000	-2.079804000

H	7.258592000	5.062122000	-0.602511000

H	8.297410000	4.763016000	0.790278000

H	10.138146000	6.079735000	-0.274224000

H	9.130103000	6.338775000	-1.709985000

H	9.393379000	10.260169000	1.480943000

H	8.393576000	10.156109000	0.006042000

H	7.780348000	9.495484000	1.517893000

H	6.671588000	7.457283000	0.099610000

H	-11.289148000	4.508645000	-1.826538000

H	-12.782370000	4.513029000	-0.891459000

H	-11.621405000	6.583925000	-0.351270000

H	-11.533907000	5.540561000	1.055327000

H	-9.467122000	6.861831000	1.864870000

H	-9.541166000	7.874507000	0.443274000

**Table S9.** Coordinates of atoms for GABA-DFO calculated structure

C	-8.574872000	4.247117000	-0.604681000

N	-9.234784000	3.244726000	-1.242037000

C	-8.810732000	1.850900000	-1.199114000

C	-9.627132000	1.008675000	-0.207285000

C	-9.262929000	-0.483882000	-0.217267000

C	-7.844224000	-0.793306000	0.281246000

C	-7.569287000	-2.298523000	0.308424000

N	-6.218265000	-2.614672000	0.764814000

C	-5.936464000	-3.643816000	1.630116000

O	-5.214841000	-2.261197000	-0.159079000

C	-4.480883000	-4.057089000	1.763988000

O	-6.832164000	-4.186049000	2.279949000

C	-4.119304000	-5.157839000	0.752592000

C	-2.754808000	-5.773620000	1.059701000

N	-2.038036000	-6.159965000	-0.025445000

C	-0.725567000	-6.788756000	0.069220000

O	-2.360165000	-5.938767000	2.217649000

C	0.434437000	-5.793760000	-0.068721000

C	1.804001000	-6.478057000	0.013360000

C	2.972286000	-5.493056000	-0.110673000

C	4.332610000	-6.195074000	-0.027878000

N	5.467628000	-5.278761000	-0.085777000

O	5.565425000	-4.411686000	1.018494000

C	6.045072000	-4.869106000	-1.260526000

C	7.025827000	-3.711681000	-1.180854000

O	5.786724000	-5.445080000	-2.319704000

C	6.310660000	-2.354150000	-1.285589000

C	7.291812000	-1.191632000	-1.146355000

N	6.994462000	-0.093134000	-1.886315000

O	8.272496000	-1.253703000	-0.399422000

C	7.739173000	1.157224000	-1.794293000

C	7.088166000	2.182419000	-0.856285000

C	7.873032000	3.497829000	-0.792891000

C	7.233783000	4.529200000	0.144462000

C	8.021783000	5.840364000	0.183647000

N	7.412835000	6.833302000	1.063629000

C	8.135836000	7.634668000	1.915106000

C	7.401492000	8.824712000	2.496355000

O	9.300198000	7.357875000	2.205826000

O	6.205663000	7.363840000	0.568011000

O	-7.518229000	4.067233000	0.009254000

C	-9.232648000	5.621276000	-0.687485000

C	-8.213141000	6.740278000	-0.928935000

C	-8.837815000	8.134241000	-0.860354000

N	-7.825408000	9.167323000	-1.127615000

H	-10.114148000	3.458321000	-1.690522000

H	-8.897554000	1.428778000	-2.207301000

H	-7.753239000	1.857842000	-0.929091000

H	-9.493343000	1.420263000	0.801240000

H	-10.691582000	1.116068000	-0.450327000

H	-9.985020000	-1.022597000	0.409342000

H	-9.386746000	-0.880759000	-1.234542000

H	-7.102250000	-0.309119000	-0.364105000

H	-7.706173000	-0.384928000	1.290451000

H	-8.242399000	-2.808844000	0.999228000

H	-7.711886000	-2.731738000	-0.690666000

H	-4.879528000	-1.403340000	0.149205000

H	-3.811348000	-3.203691000	1.639413000

H	-4.357701000	-4.453686000	2.773097000

H	-4.860019000	-5.965291000	0.816250000

H	-4.154170000	-4.766953000	-0.268315000

H	-2.398058000	-5.945629000	-0.944212000

H	-0.678588000	-7.288897000	1.039795000

H	-0.658576000	-7.557797000	-0.707986000

H	0.342706000	-5.261887000	-1.024714000

H	0.345972000	-5.038773000	0.722351000

H	1.883809000	-7.019045000	0.966513000

H	1.880094000	-7.237292000	-0.777444000

H	2.909076000	-4.956359000	-1.065494000

H	2.911817000	-4.741470000	0.684990000

H	4.418116000	-6.763556000	0.904640000

H	4.463927000	-6.889623000	-0.859847000

H	6.162318000	-4.864171000	1.637828000

H	7.600268000	-3.735517000	-0.254089000

H	7.713749000	-3.835618000	-2.021178000

H	5.753974000	-2.284962000	-2.225669000

H	5.583294000	-2.263204000	-0.470247000

H	6.151908000	-0.102776000	-2.443259000

H	8.741685000	0.904657000	-1.440683000

H	7.834799000	1.575172000	-2.802503000

H	6.062083000	2.380749000	-1.193148000

H	7.011381000	1.743933000	0.146583000

H	8.901275000	3.293981000	-0.463796000

H	7.952481000	3.923452000	-1.802806000

H	6.207628000	4.734833000	-0.185038000

H	7.167104000	4.121247000	1.160849000

H	9.030278000	5.682091000	0.569610000

H	8.104504000	6.267174000	-0.825064000

H	7.145293000	9.540311000	1.710142000

H	6.466815000	8.523592000	2.976682000

H	8.054160000	9.301264000	3.227608000

H	5.507650000	6.875264000	1.033976000

H	-10.010325000	5.634301000	-1.459415000

H	-9.740599000	5.789411000	0.272118000

H	-7.417992000	6.652995000	-0.179397000

H	-7.738085000	6.610273000	-1.908829000

H	-9.618503000	8.223910000	-1.626368000

H	-9.343101000	8.253154000	0.114226000

H	-8.270904000	10.082725000	-1.102861000

H	-7.157189000	9.176971000	-0.358000000

**Table S10.** Coordinates of atoms for DFO-Suc-SS02 calculated structure

O	10.749710000	-1.402100000	6.955451000

O	13.787486000	-0.442222000	-0.712821000

H	15.843724000	-0.844340000	-2.171082000

N	14.000070000	-0.323279000	-2.971605000

C	15.221877000	-1.113144000	-3.030866000

C	15.920175000	-0.743311000	-4.346035000

O	15.263142000	-0.393605000	-5.329147000

N	17.263837000	-0.862704000	-4.358450000

C	13.366065000	-0.062780000	-1.809008000

C	14.891675000	-2.626114000	-2.964136000

C	12.057196000	0.747800000	-1.932867000

C	12.245528000	2.207381000	-1.450794000

N	10.981038000	0.033214000	-1.237931000

H	11.753763000	0.778794000	-2.983661000

C	10.694866000	0.044780000	0.087556000

O	11.170533000	0.816147000	0.921849000

C	9.660407000	-1.002616000	0.541994000

N	8.711878000	-0.296175000	1.397169000

C	10.351363000	-2.153901000	1.306695000

H	9.093192000	-1.406807000	-0.300449000

C	7.384467000	-0.532774000	1.395371000

O	6.842160000	-1.390378000	0.687340000

C	6.546381000	0.340432000	2.338807000

C	6.201449000	-0.384649000	3.676867000

N	5.334585000	0.686469000	1.612257000

H	7.077886000	1.268114000	2.561099000

H	13.637761000	-0.011298000	-3.864683000

H	17.765817000	-0.667383000	-5.212913000

H	17.794106000	-1.094827000	-3.533028000

H	14.159340000	-2.741385000	-2.158429000

H	14.389184000	-2.914171000	-3.895926000

H	12.605303000	2.193287000	-0.422347000

H	11.256259000	2.676639000	-1.443005000

H	10.520055000	-0.677827000	-1.788845000

H	9.104656000	0.432521000	1.979989000

H	10.882311000	-1.719570000	2.162371000

H	9.558458000	-2.790914000	1.713879000

H	5.495283000	0.271068000	4.194299000

H	5.655020000	-1.296557000	3.424778000

C	16.077301000	-3.554233000	-2.678577000

C	15.619249000	-5.005008000	-2.477163000

C	16.720101000	-5.945934000	-1.965296000

N	16.283344000	-7.319589000	-1.679036000

C	14.524913000	3.188548000	-1.971297000

C	15.398088000	3.903172000	-2.794314000

C	13.187265000	2.991806000	-2.340872000

C	14.944248000	4.434030000	-4.003337000

C	12.743045000	3.532940000	-3.556577000

C	13.612418000	4.247205000	-4.382345000

H	16.814174000	-3.504823000	-3.489787000

H	16.589807000	-3.208546000	-1.769263000

H	14.790566000	-5.024593000	-1.757264000

H	15.215854000	-5.399974000	-3.420456000

H	17.544597000	-5.984021000	-2.689830000

H	17.143994000	-5.527372000	-1.042030000

H	15.474928000	-7.270268000	-1.050809000

H	15.921632000	-7.727353000	-2.541011000

H	14.883120000	2.779885000	-1.029956000

H	16.430441000	4.047559000	-2.488974000

H	15.620852000	4.992600000	-4.643322000

H	11.705650000	3.400401000	-3.854686000

H	13.248989000	4.662470000	-5.317921000

C	7.393109000	-0.686874000	4.564465000

C	7.947483000	0.331129000	5.376271000

C	7.959539000	-1.980519000	4.600343000

C	9.072962000	0.062531000	6.160452000

C	9.082533000	-2.227071000	5.399075000

C	9.647165000	-1.209120000	6.168441000

C	7.312859000	1.702942000	5.482138000

C	7.360495000	-3.140318000	3.830400000

C	4.637428000	1.825186000	1.844518000

O	4.980041000	2.650037000	2.699083000

C	3.420963000	2.041955000	0.953301000

C	2.250471000	2.658474000	1.723253000

C	1.197764000	3.239045000	0.783840000

O	1.510947000	3.837589000	-0.250232000

N	-0.089924000	3.063581000	1.175075000

C	-1.230161000	3.626980000	0.462451000

C	-1.799775000	4.885709000	1.129433000

C	-3.011248000	5.447163000	0.375346000

C	-3.611205000	6.683516000	1.054001000

C	-4.802762000	7.298880000	0.309347000

N	-5.957904000	6.411871000	0.201823000

O	-6.477935000	5.969657000	1.429790000

C	-6.292157000	5.708789000	-0.925258000

O	-5.730718000	5.938890000	-1.999346000

C	-7.404846000	4.680822000	-0.795524000

C	-8.802241000	5.321145000	-0.739729000

C	-9.889185000	4.260335000	-0.571599000

O	-9.716013000	3.261157000	0.132141000

N	-11.046600000	4.502243000	-1.237230000

C	-12.222897000	3.648550000	-1.117439000

C	-13.216977000	4.126505000	-0.050721000

C	-14.436588000	3.203631000	0.062688000

C	-15.442355000	3.678176000	1.116812000

C	-16.650314000	2.748581000	1.305545000

N	-17.479680000	2.614249000	0.119013000

O	-18.061021000	3.836288000	-0.265539000

C	-17.598658000	1.626673000	-0.798148000

O	-18.241842000	1.863091000	-1.840482000

C	-16.953409000	0.283272000	-0.509647000

C	-17.581328000	-0.830319000	-1.347143000

C	-16.747243000	-2.107391000	-1.298700000

O	-15.627992000	-2.137417000	-0.776518000

N	-17.274407000	-3.215877000	-1.883997000

C	-18.594168000	-3.392654000	-2.482935000

C	-19.628425000	-3.996331000	-1.521549000

C	-20.988546000	-4.211304000	-2.200375000

C	-22.095961000	-4.727988000	-1.268098000

C	-21.854918000	-6.152997000	-0.760477000

N	-22.952973000	-6.634637000	0.073621000

O	-23.014504000	-6.015180000	1.337682000

C	-23.466171000	-7.907182000	-0.014823000

C	-24.346296000	-8.353647000	1.133722000

O	-23.242991000	-8.610567000	-1.000807000

C	11.312460000	-3.001284000	0.463086000

C	11.891595000	-4.165833000	1.282330000

N	12.787385000	-5.033950000	0.514419000

C	12.507562000	-6.343351000	0.177656000

N	11.154650000	-6.654327000	0.101940000

N	13.484855000	-7.175796000	-0.004133000

H	13.776606000	-4.891921000	0.662705000

H	10.956611000	-7.539574000	-0.344817000

H	10.546497000	-5.918864000	-0.235799000

H	13.127627000	-8.062836000	-0.358661000

H	11.041801000	-2.321227000	6.875881000

H	5.020770000	0.010833000	0.927279000

H	9.507929000	0.839036000	6.782505000

H	9.516223000	-3.224104000	5.423165000

H	7.011311000	2.122803000	4.519725000

H	6.404844000	1.658747000	6.097690000

H	7.996087000	2.408207000	5.961944000

H	7.226701000	-2.919818000	2.767937000

H	7.994894000	-4.026337000	3.913040000

H	6.372516000	-3.407541000	4.225280000

H	3.711028000	2.738399000	0.157986000

H	3.121193000	1.108579000	0.466438000

H	1.808091000	1.929933000	2.410288000

H	2.638731000	3.480653000	2.335655000

H	-0.265702000	2.588628000	2.049023000

H	-0.887328000	3.854674000	-0.549494000

H	-2.007334000	2.857647000	0.387795000

H	-2.085733000	4.648944000	2.163012000

H	-1.009015000	5.644331000	1.188400000

H	-2.719676000	5.698488000	-0.653170000

H	-3.778276000	4.665772000	0.294982000

H	-3.921643000	6.432753000	2.075064000

H	-2.846882000	7.465873000	1.143664000

H	-5.127482000	8.215535000	0.815216000

H	-4.537540000	7.555813000	-0.717681000

H	-7.089376000	6.671258000	1.711232000

H	-7.325447000	4.043034000	-1.678957000

H	-7.267134000	4.063088000	0.093105000

H	-8.871540000	5.990703000	0.125635000

H	-8.982867000	5.929919000	-1.631278000

H	-11.133129000	5.361820000	-1.760433000

H	-12.711664000	3.601683000	-2.096432000

H	-11.864520000	2.645269000	-0.874089000

H	-12.698361000	4.180012000	0.914591000

H	-13.544221000	5.146550000	-0.290892000

H	-14.932611000	3.139619000	-0.914838000

H	-14.099292000	2.187223000	0.308898000

H	-14.947911000	3.759276000	2.092792000

H	-15.803354000	4.680908000	0.863380000

H	-16.325507000	1.745884000	1.587927000

H	-17.289299000	3.126532000	2.111157000

H	-18.417616000	3.580503000	-1.149009000

H	-15.883814000	0.345503000	-0.736832000

H	-17.023671000	0.039771000	0.553306000

H	-18.601509000	-1.033714000	-1.005843000

H	-17.672469000	-0.498305000	-2.387005000

H	-16.688561000	-4.037499000	-1.800311000

H	-18.945038000	-2.429494000	-2.861870000

H	-18.477115000	-4.043913000	-3.356152000

H	-19.233151000	-4.943989000	-1.136697000

H	-19.746223000	-3.332166000	-0.655359000

H	-21.322086000	-3.259624000	-2.633290000

H	-20.870659000	-4.904907000	-3.044359000

H	-22.197480000	-4.050257000	-0.411156000

H	-23.053545000	-4.710481000	-1.801831000

H	-21.784647000	-6.856200000	-1.592464000

H	-20.926688000	-6.215700000	-0.179408000

H	-23.704646000	-5.337532000	1.250879000

H	-25.148675000	-7.636942000	1.326833000

H	-24.772236000	-9.323285000	0.876435000

H	-23.762115000	-8.441326000	2.054092000

H	10.786375000	-3.401744000	-0.414004000

H	12.136987000	-2.386075000	0.085218000

H	12.449192000	-3.764166000	2.136094000

H	11.079166000	-4.770727000	1.697692000

**Figure S22.** The calculated structure of Fe(DFO) complex

**Table S11.** Coordinates of atoms for the calculated structure of Fe(DFO) complex

Fe	0.030168000	-0.617616000	-0.000505000

N	-7.772487000	-4.489788000	-0.196041000

C	-7.144855000	-3.170217000	-0.370116000

C	-5.842941000	-3.085874000	0.431894000

C	-5.136298000	-1.723578000	0.357343000

C	-4.597136000	-1.370614000	-1.034989000

C	-3.828011000	-0.045453000	-1.092751000

N	-2.581639000	-0.073181000	-0.327531000

C	-2.258335000	0.542318000	0.794653000

O	-1.619646000	-0.878752000	-0.889174000

C	-3.236019000	1.358281000	1.602693000

O	-1.058597000	0.390680000	1.243745000

C	-2.797611000	2.816345000	1.786088000

C	-2.790126000	3.598055000	0.472088000

N	-2.192033000	4.813506000	0.532958000

C	-1.970448000	5.654930000	-0.641743000

O	-3.292939000	3.154036000	-0.564862000

C	-0.716644000	5.296996000	-1.460835000

C	0.602167000	5.325632000	-0.660948000

C	0.933497000	4.002973000	0.061165000

C	1.618551000	2.983921000	-0.864621000

N	1.469560000	1.599874000	-0.404346000

O	0.411585000	0.931464000	-0.986603000

C	2.120632000	0.969698000	0.559316000

C	3.315982000	1.598530000	1.243017000

O	1.705825000	-0.195909000	0.914643000

C	4.622180000	1.711110000	0.420487000

C	5.602544000	0.538091000	0.350434000

N	5.194891000	-0.676473000	0.787844000

O	6.734868000	0.735019000	-0.101829000

C	6.072636000	-1.848216000	0.705858000

C	6.107959000	-2.533530000	-0.676269000

C	4.928286000	-3.473354000	-0.984537000

C	3.570592000	-2.812775000	-0.746104000

C	2.345565000	-3.643770000	-1.141521000

N	1.145659000	-2.985153000	-0.629282000

C	0.563347000	-3.197385000	0.542242000

C	0.734421000	-4.486007000	1.295922000

O	-0.173346000	-2.272114000	1.029568000

O	0.870647000	-1.770605000	-1.237472000

H	-8.083943000	-4.579071000	0.770443000

H	-8.623942000	-4.529340000	-0.753337000

H	-6.936437000	-3.043226000	-1.438915000

H	-7.798657000	-2.329443000	-0.077575000

H	-6.069791000	-3.305557000	1.484263000

H	-5.163915000	-3.878387000	0.091294000

H	-5.826625000	-0.936404000	0.692070000

H	-4.303853000	-1.726509000	1.071920000

H	-3.942613000	-2.172225000	-1.395786000

H	-5.419449000	-1.288180000	-1.754869000

H	-3.534507000	0.174366000	-2.123531000

H	-4.425819000	0.794739000	-0.742122000

H	-4.235944000	1.321952000	1.174618000

H	-3.288020000	0.886587000	2.590071000

H	-3.482215000	3.310257000	2.485306000

H	-1.803990000	2.855272000	2.244219000

H	-1.772455000	5.097700000	1.406592000

H	-2.856617000	5.579395000	-1.277023000

H	-1.899761000	6.688467000	-0.288986000

H	-0.860886000	4.311555000	-1.920046000

H	-0.663930000	6.016560000	-2.286484000

H	1.432089000	5.580750000	-1.331833000

H	0.560541000	6.137988000	0.075100000

H	1.581786000	4.203897000	0.920747000

H	0.021222000	3.550399000	0.454070000

H	1.169656000	2.989165000	-1.859703000

H	2.678842000	3.217116000	-0.985336000

H	3.037062000	2.607638000	1.559050000

H	3.483960000	1.033421000	2.163206000

H	4.407813000	1.983071000	-0.618414000

H	5.212058000	2.539566000	0.822439000

H	4.233277000	-0.801401000	1.070653000

H	7.077956000	-1.501920000	0.955867000

H	5.761738000	-2.553866000	1.482756000

H	6.175147000	-1.749299000	-1.438906000

H	7.039750000	-3.108317000	-0.740852000

H	5.009837000	-3.810895000	-2.025742000

H	5.006621000	-4.373843000	-0.360291000

H	3.460001000	-2.600041000	0.322127000

H	3.516787000	-1.851649000	-1.265070000

H	2.241672000	-3.731940000	-2.226753000

H	2.394339000	-4.651409000	-0.723189000

H	-0.132279000	-4.612034000	1.946432000

H	0.815953000	-5.350672000	0.635699000

H	1.628727000	-4.444882000	1.927018000

**Figure S23.** The calculated structure of Fe(5-ALA-DFO) complex

**Table S12.** Coordinates of atoms for the calculated structure of Fe(5-ALA-DFO) complex

N	-4.582468000	-1.806322000	0.132765000

C	-3.185684000	-1.682854000	0.553027000

C	-2.553985000	-3.040175000	0.872782000

C	-2.435410000	-4.023297000	-0.310376000

C	-1.659183000	-3.542700000	-1.550457000

C	-0.131820000	-3.464139000	-1.416451000

N	0.325486000	-2.413935000	-0.515908000

C	1.168514000	-2.484314000	0.507328000

C	1.775534000	-3.788491000	0.965218000

C	3.113783000	-4.112464000	0.254749000

C	4.316880000	-3.943273000	1.191900000

N	5.561751000	-3.869932000	0.626724000

C	6.001531000	-4.004518000	-0.757911000

C	6.938432000	-2.865072000	-1.207904000

C	6.248846000	-1.576008000	-1.686653000

O	-0.165930000	-1.172804000	-0.833352000

O	1.478432000	-1.397989000	1.104338000

O	4.186375000	-3.897403000	2.409595000

C	5.294216000	-0.934700000	-0.674580000

C	4.648430000	0.361513000	-1.184853000

N	3.390310000	0.617384000	-0.491366000

O	2.323503000	-0.047364000	-1.046658000

C	3.165189000	1.309997000	0.609819000

O	1.971487000	1.350118000	1.081834000

C	4.249256000	2.073228000	1.329355000

C	3.963480000	3.581744000	1.320813000

C	4.205140000	4.188149000	-0.062925000

O	4.987224000	3.668160000	-0.857917000

N	3.564983000	5.351895000	-0.380969000

C	2.577930000	6.112136000	0.376180000

C	1.154454000	6.058530000	-0.203597000

C	0.605183000	4.639798000	-0.414670000

C	-0.924093000	4.622621000	-0.538079000

C	-1.512739000	3.265872000	-0.959217000

N	-0.941723000	2.121135000	-0.260172000

O	0.088623000	1.507774000	-0.953109000

C	-1.306827000	1.566526000	0.884553000

O	-0.661809000	0.544677000	1.296600000

C	-2.475243000	2.081946000	1.676202000

N	-11.662591000	0.584676000	-0.372950000

C	-9.250768000	-0.179534000	-0.362818000

C	-7.969834000	-0.357064000	0.432227000

C	-6.863829000	-1.077894000	-0.334319000

C	-5.487371000	-0.804681000	0.271680000

O	-5.232287000	0.263123000	0.831430000

C	-10.498289000	0.236992000	0.424778000

O	-9.305322000	-0.350894000	-1.568333000

Fe	0.836037000	0.141979000	0.095326000

H	-4.850512000	-2.611912000	-0.412638000

H	-2.588665000	-1.162450000	-0.203046000

H	-3.172445000	-1.055845000	1.445975000

H	-1.563922000	-2.835968000	1.288653000

H	-3.133143000	-3.526933000	1.668232000

H	-1.984731000	-4.955132000	0.058821000

H	-3.443083000	-4.315356000	-0.641099000

H	-1.853817000	-4.243819000	-2.371914000

H	-2.021233000	-2.566675000	-1.884480000

H	0.274200000	-4.418113000	-1.072207000

H	0.297460000	-3.250634000	-2.402841000

H	1.978658000	-3.687397000	2.033982000

H	1.069423000	-4.611469000	0.837921000

H	3.117614000	-5.155060000	-0.087459000

H	3.231625000	-3.498755000	-0.642994000

H	6.289667000	-3.805803000	1.328163000

H	6.524168000	-4.964127000	-0.877664000

H	5.124066000	-4.049074000	-1.407590000

H	7.630050000	-2.638335000	-0.384784000

H	7.561351000	-3.247463000	-2.025907000

H	7.026297000	-0.855364000	-1.973769000

H	5.688945000	-1.796152000	-2.606618000

H	4.478518000	-1.623263000	-0.446920000

H	5.813037000	-0.744807000	0.273594000

H	5.292129000	1.238173000	-1.093844000

H	4.366543000	0.266582000	-2.237226000

H	4.247244000	1.717631000	2.364882000

H	5.233483000	1.883743000	0.903276000

H	2.943947000	3.747824000	1.671834000

H	4.634316000	4.086720000	2.027795000

H	3.790380000	5.674291000	-1.314382000

H	2.570259000	5.736252000	1.400702000

H	2.909308000	7.157003000	0.435466000

H	0.508013000	6.615966000	0.488812000

H	1.123350000	6.611595000	-1.152514000

H	1.058897000	4.187499000	-1.302255000

H	0.891098000	3.991066000	0.420908000

H	-1.360927000	4.931749000	0.421214000

H	-1.257367000	5.365606000	-1.275913000

H	-2.599180000	3.263459000	-0.830204000

H	-1.304334000	3.066826000	-2.013054000

H	-2.681480000	3.137671000	1.491380000

H	-3.373395000	1.499762000	1.435296000

H	-2.252638000	1.944917000	2.736320000

H	-11.446380000	1.416511000	-0.919063000

H	-11.796483000	-0.145826000	-1.070477000

H	-7.600883000	0.639781000	0.711577000

H	-8.196217000	-0.855860000	1.383395000

H	-7.062213000	-2.154042000	-0.391833000

H	-6.863192000	-0.709916000	-1.367462000

H	-10.238484000	1.070829000	1.089978000

H	-10.749213000	-0.598055000	1.096389000

**Figure S24.** The calculated structure of Fe(GABA-DFO) complex

**Table S13.** Coordinates of atoms for the calculated structure of Fe(GABA-DFO) complex

C	-4.986302000	-3.121597000	-0.302413000

C	-3.806292000	-2.183705000	-0.578346000

C	-4.156766000	-0.730565000	-0.928694000

N	-2.964389000	0.100703000	-0.765909000

O	-1.922220000	-0.236900000	-1.609285000

C	-2.678318000	0.908475000	0.243425000

O	-1.463696000	1.292003000	0.386156000

C	-3.751014000	1.411534000	1.191520000

C	-3.246334000	2.554987000	2.111633000

C	-4.036321000	3.852571000	1.986904000

O	-4.863964000	4.176863000	2.844993000

N	-3.816231000	4.645247000	0.902689000

C	-2.853123000	4.514736000	-0.192118000

C	-1.481842000	5.172417000	0.078750000

C	-0.814109000	5.740491000	-1.186921000

C	-0.474769000	4.768095000	-2.334423000

C	0.819465000	3.952829000	-2.194689000

N	0.693539000	2.747712000	-1.378584000

O	-0.114657000	1.787213000	-1.968286000

C	1.401532000	2.348756000	-0.333070000

O	1.213316000	1.156567000	0.100194000

C	2.403957000	3.219742000	0.363559000

Fe	-0.305003000	0.319015000	-0.808451000

C	0.811597000	-3.082854000	-2.621605000

N	0.490254000	-2.098113000	-1.585476000

C	-0.079615000	-2.309036000	-0.410785000

C	-0.418424000	-3.716754000	0.031188000

C	-0.924980000	-3.805521000	1.482112000

C	-1.461191000	-5.209234000	1.751939000

N	-2.806899000	-5.403873000	1.847358000

C	-3.908847000	-4.471036000	1.616121000

C	-4.482820000	-4.498241000	0.176601000

O	0.756218000	-0.782588000	-1.910057000

O	-0.373605000	-1.288146000	0.309146000

O	-0.676390000	-6.156237000	1.867603000

N	6.416667000	0.240735000	4.476630000

C	5.288841000	1.024398000	3.947009000

C	4.884506000	0.561476000	2.543972000

C	5.987402000	0.775636000	1.486793000

C	5.490612000	0.439793000	0.087322000

O	4.912127000	1.273931000	-0.621195000

N	5.698720000	-0.839059000	-0.314026000

C	5.179232000	-1.388040000	-1.560848000

C	3.856406000	-2.147187000	-1.383498000

C	3.422035000	-2.846839000	-2.678211000

C	2.189126000	-3.756143000	-2.537695000

H	-5.651602000	-2.683966000	0.454292000

H	-5.588782000	-3.249631000	-1.209181000

H	-3.165550000	-2.137215000	0.307003000

H	-3.191544000	-2.597115000	-1.383622000

H	-4.503976000	-0.629691000	-1.961192000

H	-4.936349000	-0.341164000	-0.271324000

H	-4.622771000	1.726686000	0.610193000

H	-4.086605000	0.570308000	1.807161000

H	-3.343786000	2.254987000	3.155125000

H	-2.186995000	2.731532000	1.915955000

H	-4.358833000	5.500064000	0.924924000

H	-2.727200000	3.460852000	-0.441536000

H	-3.319557000	4.986432000	-1.063639000

H	-1.634825000	6.004541000	0.775967000

H	-0.823925000	4.458183000	0.586201000

H	-1.486827000	6.506783000	-1.594088000

H	0.099689000	6.273930000	-0.894259000

H	-1.302292000	4.079989000	-2.535499000

H	-0.351518000	5.364098000	-3.246169000

H	1.135376000	3.606130000	-3.185002000

H	1.624433000	4.568205000	-1.790989000

H	3.403242000	2.816725000	0.163125000

H	2.363817000	4.265890000	0.064764000

H	2.221269000	3.155185000	1.439437000

H	0.032990000	-3.850829000	-2.606057000

H	0.710697000	-2.537825000	-3.563090000

H	-1.189443000	-4.094231000	-0.653428000

H	0.443537000	-4.377520000	-0.091903000

H	-0.091707000	-3.648068000	2.171910000

H	-1.654664000	-3.020967000	1.673642000

H	-3.052381000	-6.379226000	1.971692000

H	-4.698275000	-4.710797000	2.334601000

H	-3.566162000	-3.468365000	1.873327000

H	-5.290929000	-5.235444000	0.122536000

H	-3.702124000	-4.848290000	-0.509555000

H	7.279280000	0.545429000	4.029455000

H	6.540150000	0.467786000	5.461306000

H	4.436055000	0.873588000	4.618446000

H	5.481975000	2.110825000	3.923404000

H	4.619603000	-0.501936000	2.582624000

H	3.986177000	1.109176000	2.237077000

H	6.293860000	1.826482000	1.476507000

H	6.865580000	0.169382000	1.734614000

H	6.144008000	-1.476139000	0.331132000

H	5.050279000	-0.550443000	-2.250453000

H	5.940811000	-2.054192000	-1.981841000

H	3.983037000	-2.898799000	-0.592634000

H	3.083122000	-1.448706000	-1.049187000

H	3.244029000	-2.102362000	-3.465458000

H	4.256547000	-3.467151000	-3.030494000

H	2.263609000	-4.337092000	-1.609983000

H	2.195016000	-4.490627000	-3.352245000

**Figure S25.** The calculated structure of Fe(DFO-Suc-SS02) complex

**Table S14.** Coordinates of atoms for the calculated structure of Fe(DFO-Suc-SS02) complex

Fe	6.108120000	-0.833461000	1.484480000

O	-12.134391000	-0.123768000	-0.069401000

O	-2.841066000	2.527031000	-2.626906000

H	-1.455830000	4.402232000	-3.029889000

N	-0.728657000	2.884695000	-1.849892000

C	-0.748860000	4.300105000	-2.202933000

C	0.656191000	4.723534000	-2.642821000

O	1.657408000	4.393050000	-2.003115000

N	0.716708000	5.503321000	-3.743481000

C	-1.821517000	2.115239000	-2.064111000

C	-1.249408000	5.156278000	-1.016208000

C	-1.716377000	0.661352000	-1.558657000

C	-1.619363000	-0.338581000	-2.736281000

N	-2.837390000	0.383594000	-0.650224000

H	-0.818625000	0.564785000	-0.944531000

C	-4.119443000	0.077781000	-0.967283000

O	-4.542379000	-0.198663000	-2.090902000

C	-5.099311000	0.062132000	0.224206000

N	-5.868110000	-1.174372000	0.121574000

C	-6.030542000	1.292003000	0.181777000

H	-4.573159000	0.022865000	1.180888000

C	-5.927882000	-2.096541000	1.106471000

O	-5.426927000	-1.940386000	2.226003000

C	-6.645140000	-3.409559000	0.760116000

C	-7.941422000	-3.609920000	1.593527000

N	-5.671103000	-4.467371000	1.001755000

H	-6.888650000	-3.446428000	-0.302769000

H	0.042916000	2.529110000	-1.284498000

H	1.615363000	5.843077000	-4.055945000

H	-0.099957000	5.737705000	-4.285892000

H	-2.142497000	4.653877000	-0.627861000

H	-0.496393000	5.128625000	-0.218924000

H	-2.508049000	-0.234192000	-3.358303000

H	-1.635894000	-1.346821000	-2.308000000

H	-2.656539000	0.599774000	0.320156000

H	-6.198865000	-1.406945000	-0.806343000

H	-6.560403000	1.285102000	-0.778892000

H	-6.787028000	1.153993000	0.963182000

H	-8.225723000	-4.659574000	1.472505000

H	-7.687348000	-3.473549000	2.647509000

C	-1.613379000	6.603617000	-1.367491000

C	-2.290651000	7.320746000	-0.192435000

C	-2.866850000	8.699965000	-0.546925000

N	-3.630066000	9.352723000	0.526031000

C	-0.413564000	0.544505000	-4.782223000

C	0.742216000	0.740538000	-5.541678000

C	-0.364008000	-0.141758000	-3.561078000

C	1.968449000	0.250558000	-5.088649000

C	0.874929000	-0.631007000	-3.120351000

C	2.035122000	-0.438480000	-3.873722000

H	-0.723090000	7.160311000	-1.686967000

H	-2.299021000	6.598894000	-2.227042000

H	-3.110841000	6.695423000	0.183983000

H	-1.578921000	7.427970000	0.638626000

H	-2.057607000	9.372503000	-0.861777000

H	-3.531543000	8.594583000	-1.415452000

H	-4.354783000	8.698972000	0.839181000

H	-3.015433000	9.471806000	1.331127000

H	-1.366041000	0.927918000	-5.138974000

H	0.682553000	1.272554000	-6.487023000

H	2.867501000	0.399826000	-5.679919000

H	0.930206000	-1.173287000	-2.179274000

H	2.985682000	-0.827342000	-3.518717000

C	-9.083050000	-2.701290000	1.174761000

C	-9.854189000	-3.030681000	0.036570000

C	-9.392481000	-1.526742000	1.899997000

C	-10.874370000	-2.171707000	-0.388032000

C	-10.418862000	-0.686370000	1.457343000

C	-11.151583000	-0.996933000	0.311269000

C	-9.655706000	-4.331717000	-0.714520000

C	-8.675924000	-1.163353000	3.184918000

C	-5.449735000	-5.482263000	0.124387000

O	-6.150941000	-5.665779000	-0.875393000

C	-4.224488000	-6.334470000	0.416160000

C	-3.105480000	-5.973432000	-0.573149000

C	-1.741202000	-6.516207000	-0.152663000

O	-1.470194000	-6.782264000	1.020904000

N	-0.843177000	-6.641033000	-1.165421000

C	0.579407000	-6.864176000	-0.927924000

C	1.361812000	-5.552353000	-0.776988000

C	2.847774000	-5.768512000	-0.470550000

C	3.604000000	-4.444974000	-0.306509000

C	5.059628000	-4.657458000	0.121180000

N	5.762194000	-3.393566000	0.316215000

O	5.283184000	-2.630036000	1.343514000

C	6.771233000	-2.882358000	-0.374955000

O	7.200022000	-1.726713000	-0.039844000

C	7.409385000	-3.593831000	-1.547315000

C	6.641474000	-3.425221000	-2.886886000

C	6.211462000	-1.981086000	-3.119896000

O	5.099532000	-1.583728000	-2.751770000

N	7.127687000	-1.172092000	-3.706482000

C	6.988487000	0.283590000	-3.715746000

C	7.518999000	0.915550000	-2.422015000

C	7.333742000	2.438017000	-2.359018000

C	7.710924000	3.042108000	-0.991240000

C	6.578588000	3.086978000	0.046558000

N	6.013988000	1.778768000	0.378307000

O	6.888710000	0.940261000	1.007670000

C	4.786589000	1.315314000	0.170528000

O	4.525621000	0.120507000	0.550582000

C	3.703757000	2.163346000	-0.452446000

C	2.818723000	2.891729000	0.591981000

C	1.655363000	2.031609000	1.059633000

O	0.938337000	1.457078000	0.220027000

N	1.373445000	1.942430000	2.377263000

C	2.101480000	2.488806000	3.528915000

C	3.364095000	1.694500000	3.881060000

C	3.984905000	2.121032000	5.217049000

C	5.421984000	1.627947000	5.466383000

C	5.643285000	0.094537000	5.517376000

N	6.167130000	-0.465060000	4.273869000

O	5.275549000	-0.510635000	3.240256000

C	7.423128000	-0.785404000	3.987736000

C	8.500462000	-0.768685000	5.039734000

O	7.698140000	-1.120901000	2.784792000

C	-5.316104000	2.635707000	0.377150000

C	-6.313742000	3.804323000	0.412795000

N	-5.681064000	5.104855000	0.645096000

C	-5.826847000	5.850219000	1.797922000

N	-6.140431000	5.116098000	2.936148000

N	-5.703335000	7.139789000	1.741847000

H	-5.563758000	5.693576000	-0.167488000

H	-6.036228000	5.627694000	3.802060000

H	-5.734432000	4.190764000	2.995961000

H	-5.722356000	7.534534000	2.682055000

H	-12.566466000	-0.458285000	-0.868088000

H	-5.059897000	-4.342854000	1.797847000

H	-11.462356000	-2.428198000	-1.266353000

H	-10.662805000	0.218352000	2.005957000

H	-8.613542000	-4.531781000	-0.978070000

H	-9.990804000	-5.182954000	-0.107796000

H	-10.243461000	-4.338253000	-1.635878000

H	-7.590178000	-1.123985000	3.066981000

H	-9.014105000	-0.190483000	3.550234000

H	-8.882994000	-1.898900000	3.972124000

H	-3.859633000	-6.189819000	1.435929000

H	-4.500840000	-7.387024000	0.304834000

H	-3.362643000	-6.304399000	-1.583700000

H	-2.998910000	-4.881083000	-0.621867000

H	-1.119545000	-6.324865000	-2.084871000

H	0.662514000	-7.461783000	-0.017036000

H	0.978611000	-7.458102000	-1.756646000

H	1.254250000	-4.960194000	-1.695278000

H	0.903774000	-4.967052000	0.030054000

H	2.944626000	-6.360659000	0.449919000

H	3.309472000	-6.363411000	-1.270455000

H	3.588544000	-3.876024000	-1.244232000

H	3.111467000	-3.825969000	0.450645000

H	5.104268000	-5.199184000	1.072469000

H	5.604239000	-5.243100000	-0.621696000

H	8.407014000	-3.158941000	-1.640214000

H	7.538829000	-4.659983000	-1.344456000

H	7.284323000	-3.786276000	-3.695658000

H	5.733436000	-4.031562000	-2.883394000

H	8.041685000	-1.555131000	-3.905271000

H	7.516219000	0.672035000	-4.592539000

H	5.925695000	0.506000000	-3.839062000

H	7.023140000	0.433571000	-1.576651000

H	8.587416000	0.681161000	-2.322720000

H	7.950009000	2.905574000	-3.136862000

H	6.295004000	2.699782000	-2.604780000

H	8.565350000	2.503492000	-0.567922000

H	8.032780000	4.081942000	-1.119764000

H	6.953425000	3.511540000	0.983629000

H	5.769908000	3.728284000	-0.304142000

H	4.123296000	2.900806000	-1.135743000

H	3.069053000	1.501925000	-1.046109000

H	3.420120000	3.278650000	1.415575000

H	2.359086000	3.740976000	0.077474000

H	0.574960000	1.352103000	2.576246000

H	1.397427000	2.475652000	4.366201000

H	2.342110000	3.541021000	3.344516000

H	3.120318000	0.629202000	3.901962000

H	4.100842000	1.809110000	3.080594000

H	4.005273000	3.217815000	5.275815000

H	3.339750000	1.788262000	6.041698000

H	6.094144000	2.047043000	4.708112000

H	5.752802000	2.038816000	6.426764000

H	6.353424000	-0.155758000	6.306204000

H	4.711630000	-0.436623000	5.736229000

H	8.696602000	0.248653000	5.391530000

H	8.234270000	-1.381719000	5.904903000

H	9.412177000	-1.165046000	4.593319000

H	-4.743143000	2.620322000	1.314080000

H	-4.595572000	2.807155000	-0.431033000

H	-6.852928000	3.848842000	-0.540145000

H	-7.066038000	3.629498000	1.188738000

**Table S15.** Coordinates of atoms for the calculated structure of the transferrin active site.

C	-0.690113000	-1.229388000	2.557411000

O	0.296980000	-1.764185000	1.853085000

O	-1.045356000	-1.607375000	3.683651000

O	-1.229460000	-0.230643000	1.878721000

Fe	-0.037279000	-0.539503000	0.397414000

C	-9.098727000	-2.314160000	1.100374000

O	-9.740447000	-3.336586000	1.221789000

C	-7.579573000	-2.247962000	1.197596000

C	-6.993044000	-2.244864000	-0.256890000

N	-7.119535000	-3.338740000	2.050675000

C	-5.487910000	-2.127213000	-0.284711000

C	-4.852488000	-0.874790000	-0.200383000

C	-4.658521000	-3.256991000	-0.364327000

C	-3.269278000	-3.146564000	-0.358177000

C	-2.620186000	-1.884951000	-0.274124000

C	-3.469149000	-0.744961000	-0.195628000

O	-1.310649000	-1.820832000	-0.335067000

O	-9.736179000	-1.163894000	0.784815000

H	-7.299059000	-1.296459000	1.669276000

C	8.833160000	-3.180001000	-1.215446000

O	9.191116000	-3.864180000	-2.155841000

C	7.490198000	-2.434573000	-1.162741000

C	6.346005000	-3.475682000	-1.024932000

N	7.521618000	-1.516160000	-0.013734000

C	4.967406000	-2.856612000	-1.002322000

C	4.244895000	-2.707805000	0.192271000

C	4.360702000	-2.380322000	-2.177570000

C	3.105277000	-1.786444000	-2.157530000

C	2.366137000	-1.628315000	-0.949661000

C	2.983273000	-2.114941000	0.233531000

O	1.173641000	-1.078815000	-1.008470000

O	9.559488000	-3.041827000	-0.101339000

H	7.369490000	-1.913089000	-2.119372000

C	-1.291910000	2.858779000	-2.019721000

C	-2.703965000	4.691426000	-2.947861000

C	-0.984205000	1.349298000	-1.869368000

C	-2.585488000	3.154390000	-2.817582000

O	-2.352565000	5.321446000	-3.927936000

O	-1.024119000	0.631581000	-2.889894000

N	-2.638873000	2.596141000	-4.153280000

O	-0.682136000	1.011087000	-0.674954000

O	-3.189130000	5.272415000	-1.839805000

O	-3.660351000	2.632805000	-2.042105000

C	2.877366000	4.772432000	3.176831000

O	1.782777000	4.739544000	3.693385000

C	3.133024000	4.823206000	1.672243000

C	3.762351000	3.474073000	1.182801000

N	1.898110000	5.203493000	0.991997000

C	2.880842000	2.277107000	1.376083000

N	2.880490000	1.561162000	2.557191000

C	1.957098000	0.615917000	2.358701000

N	1.355545000	0.660367000	1.144209000

C	1.947427000	1.728970000	0.512827000

O	3.981100000	4.743721000	3.964872000

H	3.871403000	5.617028000	1.494537000

H	-7.314982000	-3.173826000	-0.743581000

H	-7.448103000	-1.417090000	-0.815780000

H	-7.556964000	-4.199250000	1.724006000

H	-6.116676000	-3.450989000	1.910826000

H	-5.464087000	0.025989000	-0.151784000

H	-5.110632000	-4.244372000	-0.446471000

H	-2.649926000	-4.037523000	-0.432829000

H	-3.005293000	0.231706000	-0.151093000

H	-9.114458000	-0.419996000	0.758534000

H	6.454091000	-4.176391000	-1.859605000

H	6.514883000	-4.046838000	-0.103528000

H	7.784703000	-0.578294000	-0.304278000

H	6.598623000	-1.446023000	0.409077000

H	4.677318000	-3.082996000	1.119749000

H	4.883194000	-2.482387000	-3.127935000

H	2.651507000	-1.432020000	-3.080171000

H	2.448376000	-2.020644000	1.169979000

H	9.006120000	-2.413342000	0.446856000

H	-1.363330000	3.318510000	-1.032232000

H	-0.450743000	3.323703000	-2.548497000

H	-2.206503000	3.248713000	-4.802516000

H	-2.087837000	1.734330000	-4.132633000

H	-3.176504000	6.233873000	-1.991655000

H	-4.369819000	2.472100000	-2.682772000

H	3.995018000	3.614031000	0.120690000

H	4.721194000	3.311256000	1.692420000

H	2.094944000	5.262174000	-0.005942000

H	1.238241000	4.432087000	1.093629000

H	1.716902000	-0.152265000	3.080497000

H	1.667371000	2.016447000	-0.489058000

H	4.787995000	4.796612000	3.430071000

**Table S16.** Coordinates of atoms for the calculated structure of the transferrin active site without iron.

C	0.539238000	-0.974897000	-2.926039000

O	-0.319670000	-1.529554000	-2.065833000

O	0.705029000	-1.314661000	-4.095140000

O	1.159619000	0.021621000	-2.297044000

C	9.394716000	-2.053531000	-1.275691000

O	10.144439000	-2.970068000	-1.023141000

C	7.881970000	-2.145300000	-1.355733000

C	7.265459000	-1.586997000	-0.014749000

N	7.493999000	-3.508287000	-1.689277000

C	5.762362000	-1.559403000	-0.019718000

C	5.053452000	-0.483670000	-0.591585000

C	4.993558000	-2.614519000	0.506247000

C	3.606448000	-2.597153000	0.468888000

C	2.879144000	-1.506456000	-0.101909000

C	3.670443000	-0.440652000	-0.638823000

O	1.587535000	-1.535627000	-0.090269000

O	9.907895000	-0.792757000	-1.422816000

H	7.533099000	-1.484497000	-2.162834000

H	7.646937000	-2.219828000	0.799029000

H	7.670207000	-0.577309000	0.157878000

H	7.978417000	-4.133698000	-1.046914000

H	6.495755000	-3.590126000	-1.491172000

H	5.615756000	0.356135000	-1.006751000

H	5.502374000	-3.458098000	0.977615000

H	3.027553000	-3.410886000	0.896558000

H	3.150729000	0.389777000	-1.097954000

H	9.175944000	-0.182175000	-1.595834000

H	1.199747000	-1.100808000	-0.952631000

C	-8.061425000	-3.761925000	1.688270000

O	-8.478122000	-4.194271000	2.745068000

C	-6.787923000	-2.916696000	1.541665000

C	-5.532616000	-3.824870000	1.744758000

N	-6.780103000	-2.324255000	0.192417000

C	-4.235155000	-3.086464000	1.540208000

C	-3.541708000	-3.140042000	0.314882000

C	-3.704040000	-2.239772000	2.531381000

C	-2.563101000	-1.485655000	2.308825000

C	-1.867333000	-1.508776000	1.056848000

C	-2.398956000	-2.389468000	0.064129000

O	-0.817066000	-0.766228000	0.907641000

O	-8.648701000	-4.025709000	0.508048000

H	-6.789196000	-2.164698000	2.339232000

H	-5.618630000	-4.251918000	2.750952000

H	-5.609427000	-4.660029000	1.034758000

H	-7.134822000	-1.371635000	0.208538000

H	-5.814811000	-2.262997000	-0.137242000

H	-3.907114000	-3.810825000	-0.465897000

H	-4.203677000	-2.176123000	3.500167000

H	-2.158682000	-0.843295000	3.086413000

H	-1.883919000	-2.461569000	-0.886991000

H	-8.084733000	-3.492319000	-0.125783000

H	-0.759803000	-0.535999000	-0.073019000

C	2.021154000	2.953938000	1.563729000

C	2.671855000	4.804651000	3.083012000

C	1.541268000	1.482919000	1.403259000

C	2.147783000	3.366307000	3.061782000

O	1.977813000	5.802203000	3.013297000

O	1.790411000	0.693428000	2.341719000

N	0.860245000	3.377792000	3.778987000

O	0.958107000	1.259883000	0.299822000

O	4.025279000	4.897441000	3.102214000

O	3.049964000	2.520885000	3.714502000

H	3.006405000	3.030155000	1.086005000

H	1.341694000	3.624143000	1.029373000

H	0.221880000	4.000263000	3.285572000

H	0.486537000	2.433253000	3.684454000

H	4.191429000	5.852439000	3.030654000

H	2.767076000	1.627209000	3.372095000

H	0.880376000	0.316537000	0.044488000

C	-5.517752000	3.518579000	-2.906997000

O	-6.645239000	3.799088000	-2.460905000

C	-4.314297000	4.371341000	-2.359650000

C	-3.730456000	3.702706000	-1.078855000

N	-3.322239000	4.606497000	-3.421875000

C	-2.876669000	2.486453000	-1.307079000

N	-3.172483000	1.532817000	-2.264382000

C	-2.229917000	0.567687000	-2.198668000

N	-1.345906000	0.837195000	-1.244705000

C	-1.738753000	2.033721000	-0.682984000

O	-5.204221000	2.594914000	-3.733716000

H	-4.749204000	5.326308000	-2.035590000

H	-3.116424000	4.432381000	-0.533182000

H	-4.581362000	3.467501000	-0.422983000

H	-2.381724000	4.490740000	-3.045777000

H	-3.455616000	3.826851000	-4.069080000

H	-2.183604000	-0.308193000	-2.825352000

H	-1.169097000	2.469090000	0.121471000

H	-3.941706000	1.684922000	-2.952429000

**Figure S26.** Structures of SS02 peptide moieties.

**Figure S27.** Structure of transferrin **(a)** without iron atoms and **(b)** its active site with iron.

**(b)**

**(a)**

**Figure S28.** Caco-2 cell viability after 24h exposure to 25 or 100 µM de 5-ALA-DFO, GABA-DFO, DFO or SIH or 5 µM de Fe(hq). It is also included the data for HBS (Ctrl) or 1% of DMSO (Ctrl DMSO 1%). Experimental data are expressed as mean values ± SD from n = 3 independent assays performed in triplicate. Statistical analysis was performed through unidimensional ANOVA, followed by *post hoc* Tukey test.

**Figure S29.** Calculated IR spectrum for DFO

**Figure S30.** Calculated IR spectrum for GABA-DFO

**Figure S31.** Calculated IR spectrum for 5-ALA-DFO

**Calculation of chelating constants K_eq_ from thermodynamic parameters**

For the chelating processes:

Tf-Fe + H_3_L ⇌ TfH_3_ + FeL

where L = DFO, 5-ALA-DFO, GABA-DFO or DFO-Suc-SS02 the same approach was used to calculate reactions constants.

Δ_r_H = H(FeL) + H(TfH_3_) – H(Tf-Fe) – H(H_3_L) (J/mol)

Δ_r_S = S(FeL) + S(TfH_3_) – S(Tf-Fe) – S(H_3_L) (J/mol)

K_eq_ = exp(Δ_r_S/8.314 – Δ_r_H/(298.15·8.314))
